# A prediction model for ‘ICU mortality or prolonged ICU stay’ in critically unwell patients with acute pancreatitis: insights from a 2003–2020 cohort analysis using the ANZICS-CORE database

**DOI:** 10.1186/s13054-025-05590-6

**Published:** 2025-08-06

**Authors:** Karthik Venkatesh, Timothy E. Schlub, S. George Barreto, Christopher R. Andersen, Miles P. Davenport, Anthony Delaney, Billingsley Kaambwa, Shailesh Bihari, David Pilcher, Sarah C. Sasson, the P-ANZICS Collaborative

**Affiliations:** 1 Department of Intensive Care, Princess Alexandra Hospital, Woolloongabba, QLD Australia; 2 The Kirby Institute, University of New South Wales, Sydney, NSW, Australia; 3 The George Institute for Global Health, Sydney, NSW Australia; 4 Faculty of Medicine and Health, The University of Sydney, Camperdown, NSW Australia; 5 Department of Hepato-Pancreato-Biliary Surgery, Flinders Medical Centre, Bedford Park, SA Australia; 6 College of Medicine and Public Health, Flinders University, Adelaide, SA Australia; 7 Department of Intensive Care, Royal North Shore Hospital St Leonard’s, Sydney, NSW, Australia; 8 Department of Intensive Care, Flinders Medical Centre, Bedford Park, SA Australia; 9 Department of Intensive Care, Alfred Hospital, Melbourne, VIC Australia; 10 Australian and New Zealand Intensive Care Research Centre (ANZIC-RC), Monash University, Melbourne, VIC, Australia; 11 Australian and New Zealand Intensive Care Society (ANZICS) Centre for Outcomes and Resource Evaluation, Prahran, VIC Australia

**Keywords:** Severe acute pancreatitis, Critical illness, Intensive care, Length of stay, Morbidity, Prediction model development

## Abstract

**Objective:**

Critically unwell patients with acute pancreatitis (AP) are at increased risk of mortality and prolonged ICU length of stay (LOS). We quantified the frequency, risk factors and complications of prolonged ICU LOS in a large cohort of critically unwell adult patients with AP and developed a model to predict a low-risk trajectory ‘survived ICU with ICU LOS ≤7 days’ versus a high-risk trajectory ‘ICU mortality or ICU LOS > 7 days’.

**Methods:**

A retrospective cohort analysis of adult patients admitted to Australian and New Zealand ICUs with AP between 2003 and 2020 was conducted using the Australian and New Zealand Intensive Care Society Centre for Outcome Reporting and Evaluation database. Data was censored to December 2020 in order to pre-date the COVID-19 pandemic. The incidence, risk factors and outcomes related to prolonged ICU LOS in AP patients was reported. Multivariate logistic regression was used to build a prediction model for a low-risk versus high-risk outcome. Discrimination was performed with 10-fold cross validation and calibration plot analysis was reported.

**Main results:**

13,275 patients met inclusion criteria; 60% were male, with a mean age 59±18, mean APACHE III 56±26. 2860 (21.6%) had an ICU LOS > 7 days, 1022 (7.7%) died in ICU, and 3557 (26.8%) had a high-risk trajectory. Prolonged ICU LOS was associated with increased ICU mortality (OR 1.57 95% CI 1.43–1.73 *p* < 0.001), hospital mortality (OR 1.69 95% CI 1.56–1.83 *p* < 0.001), and resource use: mechanical ventilation (OR 5.99 95% CI 5.21–6.90 *p* < 0.001), inotrope/vasopressor support (OR 3.27 95% CI 2.82–3.79 *p* < 0.001) and dialysis (OR 4.12 95% CI 3.63–4.68 *p* < 0.001). Model accuracy was 79.5%, Cohen K = 0.49 and AUROC 0.827. For a high-risk trajectory, sensitivity was 0.54 and specificity 0.916. APACHE III, PaO2:FiO2 ratio and early mechanical ventilation were the most influential covariates. Prolonged ICU LOS was associated with increased rate of hospital discharge to rehabilitation or a nursing home.

**Conclusions:**

More than a quarter of ICU patients with AP have a high-risk trajectory. Prolonged ICU admissions are associated with significantly worse mortality and hospital outcomes, and increase resource use. Our prediction model, if confirmed in future studies, may present an opportunity for prognostic enrichment in patients with more severe disease.

**Supplementary Information:**

The online version contains supplementary material available at 10.1186/s13054-025-05590-6.

## Introduction

Admission to the intensive care unit (ICU) with acute pancreatitis (AP) is associated with high mortality and morbidity. We recently performed a large retrospective study of critically unwell patients with AP admitted to Australian and New Zealand ICUs between 2003 and 2020. We observed a rising annual incidence in ICU AP admissions without a trend of improvement in mortality, ICU length of stay (LOS), or costs of ICU care over 18 years [1]. Across the study period, the mean ICU LOS (7 days) was consistently higher than the median (3 days), suggesting a skewed distribution. This may indicate a substantial outlier cohort with more severe disease (e.g. necrotising complications) or persistent critical illness, who are at risk of a lengthy ICU admission. Prolonged ICU admissions are associated with increased mortality and resource use, and long-term physical and psychological sequelae in survivors [2, 3]. Current severity scores used in AP (such as the Glasgow-Imrie or BISAP scores) or for critically unwell patients (such as the APACHE or SOFA scores) predict severity through mortality risk. However they are limited in their stratification of more severe illness [4]. No scores to date have been developed to predict the risk of a prolonged LOS in ICU for AP.

Improved understanding of the contributors related to prolonged ICU LOS in AP may facilitate earlier identification of a subgroup of patients at higher risk of adverse outcomes. This may enable better resource allocation, more targeted intervention, and improved participant selection in future clinical trials. Therefore the objective of this study was to test the hypothesis that a prolonged ICU LOS in critically unwell patients with AP is associated with increased mortality and hospital resource use. The second objective was to then develop a prediction model, using routine ICU admission data, to stratify the risk of a high-risk trajectory, which we defined as a composite of ICU mortality or a prolonged LOS in ICU.

## Methods

### Study design

This was a retrospective observational study of prospectively collected data from the Australian and New Zealand Intensive Care Society (ANZICS) Adult Patient Database (APD). The ANZICS APD is a Clinical Quality Registry dataset collected by the ANZICS Centre for Outcome and Resources Evaluation (ANZICS-CORE), for the purposes of benchmarking ICU practices and outcomes throughout Australia and New Zealand. The database contains over 3·5 million de-identified patient episodes, submitted from 98% of ICUs in Australia and 67% of ICUs in New Zealand. The database collects demographic and comorbidity-related data, as well as severity of illness data from the first 24 h in ICU, together with in-ICU and in-hospital outcomes including mortality, length of stay and readmission. In 2016, the dataset was expanded to include additional information about therapies provided and complications during the ICU admission (Table S1 Supplementary Appendix) [5]. Data was collected for all APD admissions from January 2003 to December 2020. The cut-off date was set at December 2020 to mitigate the confounding effects of the COVID-19 pandemic on ICU admission practices and resource use. Ethics approval was obtained from the Alfred Hospital Human Research Ethics Committee in Melbourne, Australia (HREC 286/21). Data has been analysed and reported in accordance with the STROBE reporting guideline for observational studies and the TRIPOD reporting guideline for prediction model development studies [6, 7].

### Inclusion criteria

All adult (age ≥18) patient episodes with an admission diagnosis of ‘acute pancreatitis’ were included. The admission diagnosis is provided by the treating intensivist at the time of admission to ICU, with a corresponding APACHE III diagnostic code matched to the admission episode. Only the first ICU admission was included; any ICU readmissions were excluded from the analysis.

### Outcomes

The primary outcome was the frequency of a high-risk trajectory in patients admitted to ICU with AP. We defined a high-risk trajectory as ‘death in ICU or an ICU LOS > 7 days’, and a low-risk trajectory as ‘survived ICU with an ICU LOS ≤7 days’. The seven day cut-off was supported by prior definitions for prolonged ICU LOS [8–10]. Additionally, it represents a clinically pragmatic timepoint, and in our prior study it represented the upper quartile for LOS distribution [1]. Secondary outcomes included requirement for organ support, hospital mortality, hospital LOS, hospital discharge disposition and annual trends in ICU LOS and bed occupancy (definitions listed in the Supplementary Data).

### Statistical analysis

ICU LOS for each patient was measured in hours from time of admission into ICU to discharge from ICU. We performed a descriptive analysis comparing patients with a low-risk versus high-risk trajectory. This analysis included baseline demographics, ICU admission parameters and treatment-related variables, ICU and hospital mortality, and hospital discharge disposition. We then compared characteristics of patients with an ICU LOS ≤7 versus > 7 days (irrespective of ICU survival status) to mitigate the competing risk of death on outcomes related to ICU LOS. An additional analysis was performed between ICU survivors and ICU non-survivors (irrespective of LOS status). Risk ratios were calculated to compare the relative risk of ICU mortality, hospital mortality and need for ICU organ supports between patients with a LOS ≤ 7 versus > 7 days. We examined proportions of patients with a LOS > 7 days in the entire adult cohort in the ANZICS-CORE database across the same period, with a subgroup analysis of the medical and emergency surgical admission cohorts.

For the descriptive analysis, frequencies are reported as numbers and percentages, while averages are reported as median with interquartile range (IQR) or mean with standard deviation (SD). Tests of significance were determined using the t-test for parametric continuous variables, the Mann-Whitney U test for non-parametric continuous variables and chi-squared tests for categorical variables. A p value < 0.05 was considered significant. Odds ratios are reported with 95% confidence intervals. Descriptive analysis was performed using SPSS (version 30.0.0.0 IBM 2024).

### Development of the prediction model

The objective of the model was to facilitate early (within the first 24 h of ICU admission) stratification of a low-risk or high-risk trajectory, using routine ICU admission data. The outcomes were defined as (1) discharged alive from ICU within 7 days (low-risk outcome) or (2) died in ICU or an ICU LOS ≥7 days (high-risk outcome). The 7 day mark was defined as 168 h, with the low-risk outcome group having a LOS < 168 h, and the high-risk cohort having a LOS ≥168 h.

We selected 20 variables with a literature-supported or clinically justifiable association with an increased ICU LOS or mortality due to AP, for inclusion in the model (Table S2 Supplementary Appendix). Only variables available within the first 24 h of ICU admission were selected, consistent with the aim of early stratification of the ICU outcome. To ensure that collinear variables did not affect the performance of the model, we assessed for collinearity prior to the development of the prediction model using the variance inflation factor (VIF). The VIFs were below 2 for all variables, except for urea (2.33), creatinine (2.26) and APACHE III scores (3.71). However as these levels still demonstrated relatively low collinearity, this did not require further adjustment.

The prediction model was developed using data for all years. For each dataset, both a complete case analysis and an analysis with multiple imputation by chained equations (using the *mice* package in R [11] were performed. Ten rounds of multiple imputation were conducted. Due to the large amount of missing data, imputation provided little to no benefit in predictive capability of the model, likely due to violations of the missing at random assumption. A logistic regression model with 10-fold cross-validation was used to fit and evaluate the predictive capability of the data on the outcome using the *caret* package in R [12]. For all numerical covariates, both the original values and their logarithms were included as potential covariates. This approach accommodates highly skewed numerical variables and allows for a moderate level of adjustment to non-linearity. The evaluation of predictive capability using 10-fold cross-validation ensure that predictive measures are not inflated due to overfitting. Variable importance was assessed using a permutation-based approach on model accuracy, using package *vip* in R [13]. This metric represents the expected decrease in predictive accuracy when a given variable is excluded from the model. The variable importance results are based on 50 simulation runs. The output of the prediction model was to predict the probability of a high-risk versus a low-risk outcome.

The prediction model analyses, calculations and sensitivity analyses were performed using R Statistical Software (version 3.16 R Core Team 2021).

## Results

### Sample characteristics

**Fig. 1:**
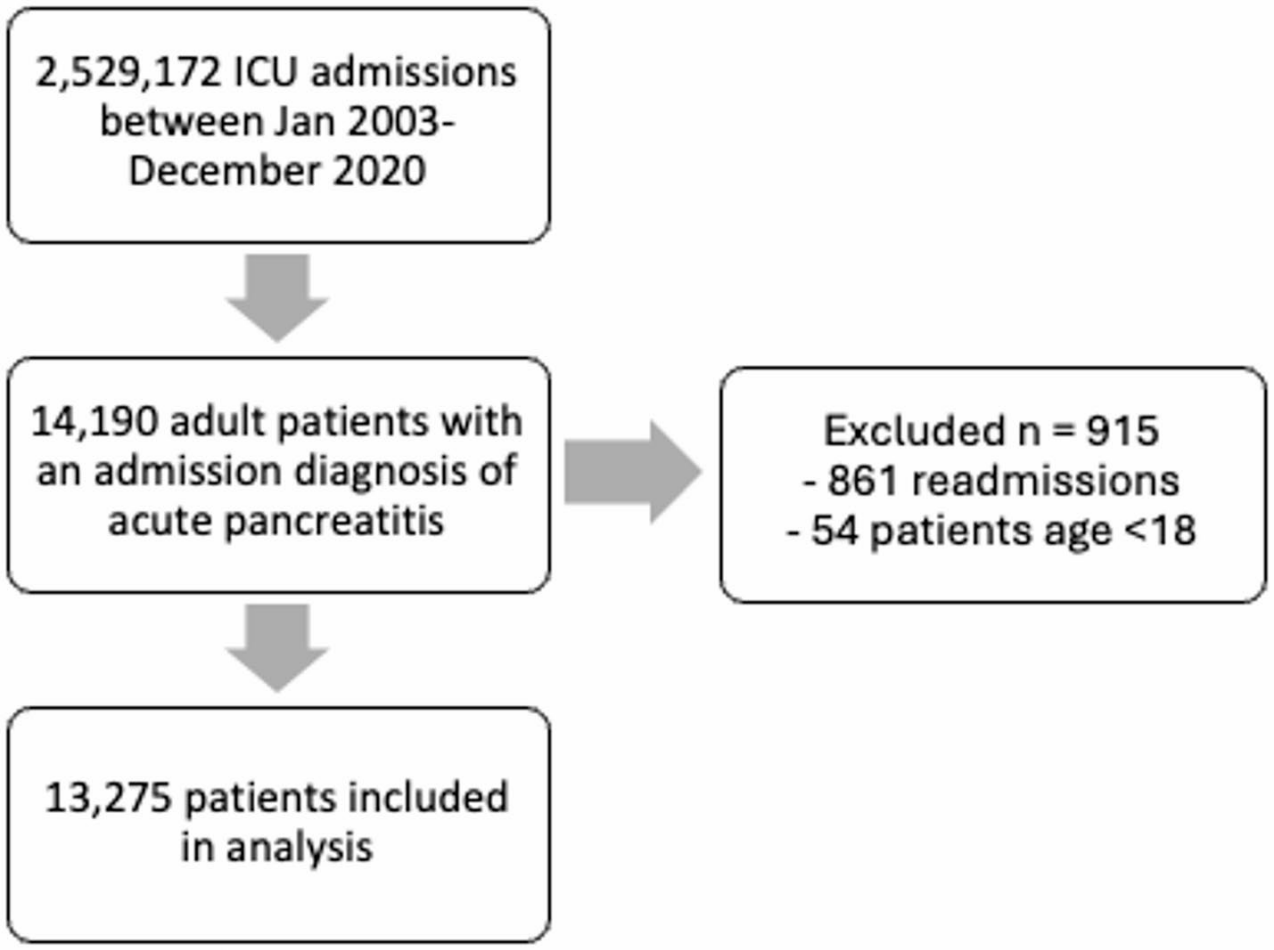
Flowchart of patient selection and inclusion into analysis

Between January 2003 and December 2020 there were 2,529,172 ICU admission episodes in 2,408,762 patients reported to the ANZICS APD. Of these, 14,190 had an admission diagnosis of acute pancreatitis; 861 readmissions and 54 patients age < 18 were excluded, leaving 13,275 patients for the analysis (Fig. 1). Median LOS across the whole cohort was 68 h (IQR 33–144). Of the 13,275 patients, 10,415 (78.4%) had an ICU LOS ≤7 days, and 2860 (21.6%) > 7 days. There were 1022 (7.7%) deaths in ICU during the study period. There were 9718 (73.2%) with an ICU LOS ≤7 days and survived (low-risk trajectory) and 3557 (26.8%) who died or had an ICU LOS greater than 7 days (high-risk trajectory).

### Low-risk versus high-risk trajectory groups

A comparison of admission characteristics, ICU treatments and outcomes between the low and high-risk trajectory groups is shown in Table 1. High-risk trajectory patients were older, more likely to be male and had higher illness severity at ICU admission. A significantly greater proportion of this group were admitted to ICU following a medical emergency response call, and there was a significantly higher incidence of organ support requirements during the admission.

### ICU LOS ≤7 days versus > 7 days cohorts (irrespective of survival status)

An analysis comparing patients with an ICU LOS ≤7 days versus > 7 days (irrespective of ICU survival status) demonstrated a similar pattern of results (Table S3 Supplementary Data). Patients with a prolonged LOS had significantly higher illness severity at admission (APACHE III 69 ± 25 vs. 59± 18 *p* < 0.001), were more likely to be male, and had a significantly higher frequency of admission following a medical emergency response call. Prolonged LOS was associated with an increased risk of ICU and hospital mortality (OR 1.57 95% CI 1.43–1.73 *p* < 0.001 and OR 1.69 95% CI 1.56–1.83 *p* < 0.001). Additionally, it was associated with an increased likelihood of requiring any organ support during the ICU admission: mechanical ventilation (OR 5.99 95% CI 5.21–6.90 *p* < 0.001), inotrope/vasopressor support (OR 3.27 95% CI 2.82–3.79 *p* < 0.001) and renal-replacement therapy (OR 4.12 95% CI 3.63–4.68 *p* < 0.001).

**Table 1.** Baseline characteristics comparing the low-risk trajectory vs. high-risk trajectory cohorts

	Total n = 13275	Low-risk trajectory n = 9718 (73.2%)	High-risk trajectory n = 3557 (26.8%)	P
Mean age (SD)	59 (18)	58 (18)	61 (17)	<0.001
Mean APACHE III (SD)	56 (26)	48 (20)	76 (30)	<0.001

	**N (%)**	**N (%)**	**N (%)**	
Male sex	7911 (60)	5632 (58)	2279 (64)	<0.001
Emergency response call	1941 (18)	1271 (16)	669 (23)	<0.001
Clinical status within first 24 hours of ICU admission				
*Invasive ventilation, n (%)*	557 (15)	179 (6)	378 (41)	<0.001
*Acute kidney injury, n (%)*	1420 (11)	529 (6)	891 (26)	<0.001
*Urea, mean (SD)*	9.4 (8.3)	8.3 (7.8)	12.6 (8.6)	<0.001
*Lactate, mean (SD)*	2.6 (2.8)	2.1 (2.1)	4 (3.8)	<0.001
*P:F ratio, mean (SD)*	255 (137)	283 (136)	199 (119)	<0.001
Interventions received during ICU admission, n (% of total datapoints available)^*a*^				
*Invasive ventilation*^a^	726 (24)	202 (9)	524 (65)	<0.001
*Inotropes*^a^	917 (32)	441 (20)	476 (65)	<0.001
*Renal replacement therapy*^a^	377 (14)	69 (3)	308 (42)	<0.001
Chronic conditions, n (% of total))				
*Chronic respiratory*	596 (4.5)	441 (4.5)	155 (4.3)	0.65
*Chronic cardiovascular*	944 (7.1)	704 (7.2)	240 (6.7)	0.34
*Chronic liver*	312 (2.4)	218 (2.2)	94 (2.7)	0.15
*Chronic renal*	368 (2.8)	247 (2.5)	121 (3.4)	0.007
*Immunosuppressive treatment*	337 (2.5)	259 (2.7)	78 (2.2)	0.15
Outcomes				
*ICU mortality, n (%)*	1022 (7.7)	0 (0)	1022 (28.9)	N/A
*Hospital mortality, n(%)*	1480 (11.2)	290 (3.0)	1190 (33.7)	<0.001
*ICU LOS (hours), median IQR 1-3)*	68 (33-144)	49 (26-88)	261 (180-465)	<0.001
*Hospital LOS (hours), median (IQR 1-3)*	273 (148-512)	230 (137-388)	512 (272-997)	<0.001

APACHE = (Acute Physiology and Chronic Health Evaluation), P:F Ratio = Ratio of arterial oxygen tension (mmHg) to fraction of inspired oxygen (as a decimal), RRT = Renal replacement therapy.^a^These variables were only available from 2017 onwards. Datapoints on mechanical ventilation (n = 3102), inotropes (n = 2902), renal replacement therapy (n = 2811)

A comparative analysis of patients who survived ICU against those who died in ICU is demonstrated in Table S4 Supplementary Appendix. The median ICU LOS was similar between ICU survivors and non-survivors, but non-survivors had substantially broader interquartile ranges and a higher SD.

### Hospital discharge disposition

Significantly fewer patients in the prolonged LOS cohort were discharged directly home (80% vs. 67% *p* < 0.001), and a significantly greater proportion were discharged to rehabilitation/another acute facility (14% vs. 25% *p* < 0.001) or a nursing home (3% vs. 9% *p* < 0.001) (Table S3 Supplementary Appendix).

### Prediction model

There was substantial variation in the dataset completeness between the covariates as several of these were only captured from 2016. Proportions of missing data for each covariate are reported in Supplementary Fig S1. Given the high proportion of missing values in potentially important predictive covariates, models were developed from two datasets. The first dataset used datapoints from the entire study period, but excluded covariates with very high missing rates (frailty, lactate, BMI, and invasively ventilated on day 1– all missing at rates greater than 60%). The second dataset only included data from 2017 onwards. Two models were developed, Model 1 using the whole dataset (*n* = 5,882), and Model 2 using only data from 2017 onwards (*n* = 542) (Table 2).

**Fig. 2:**
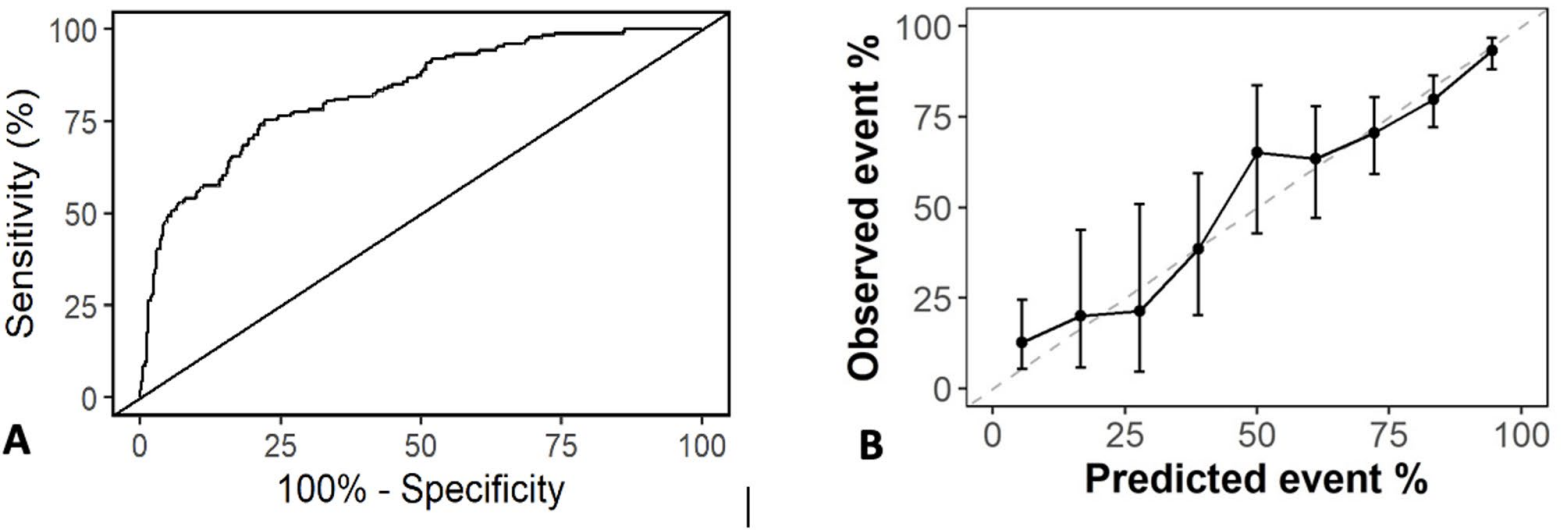
(**A**) Receiver operator characteristics curve for prediction Model 2 AUC 0.827. (**B**) Calibration plot analysis for prediction model with 95% confidence intervals Brier 0.148

Although performance was comparable between the models, Model 2 had the most effective metrics with 79.5% prediction accuracy, higher than the 59.7% accuracy expected by random chance. The Cohen’s Kappa value of 0.49 suggests that approximately half of the incorrect predictions made by a proportional model would now be correctly classified using the prediction model. For a high-risk outcome prediction, sensitivity was 0.54, while specificity was 0.916. The area under the ROC curve (AUROC) was 0.829, while calibration plot analysis demonstrated consistent accuracy with a Brier score of 0.148, though with wider confidence intervals at the lower end (Fig. 2). Precision to predict a low-risk trajectory was 80.8%, while precision to predict a high-risk trajectory was 75.2%. The F1 score for a low-risk trajectory for 85.9% and 62.9% for a high-risk trajectory. For Model 2, the individual covariates and their relevant estimate size, standard error, odds ratios and importance are reported in Table 3. The covariates with greatest predictive capability were the APACHE III score, P:F ratio, and whether the patient was invasively ventilated on day one.

**Table 2:** Multivariate prediction models for a high-risk versus low-risk trajectory

	Model 1	Model 2
Data used*	All data	Data from 2017 onwards
Variables removed	Frailty Lactate BMI Invasive ventilation day 1	Nil
Imputation	No	No
Sample size	5,882	542
High-risk trajectory, n (%)	1875 (31.9%)	174 (32.1%)
Expected Accuracy by chance	59.8%	59.7%
Prediction model accuracy	77.7%	79.5%
Cohen’s Kappa	0.446	0.490
Sensitivity (high-risk trajectory)	51%	53.9%
Specificity (high-risk trajectory)	90.2%	91.6%
AUROC	82.3	82.7

* In the complete dataset, the variables with missing values exceeding 20% were frailty (83.5%), lactate (81.5%), BMI (77.3%), invasively ventilated on day 1 (71.3%), and PF ratio (30.5%). For data from 2017 onwards, the variables with missing values exceeding 20% were BMI (56.3%), frailty (48.1%), lactate (41.1%), and PF ratio (32.7%)

As a sensitivity analysis for outcome skewness, two additional models were generated using the Model 2 dataset and covariates. The first of these, Model 3, set the outcome LOS cut off at 5 days, and the second, Model 4, set the outcome LOS at 4 days (both *n* = 542). Compared to Model 2, they both had reduced accuracy, specificity and precision, with a slight improvement in sensitivity. A further model (Model 5) was generated (with the original 7-day LOS cut off) from the 2017-onwards dataset using only the three most influential covariates (*n* = 2264). This demonstrated very comparable predictive performance to Model 2. The comparison of prediction metrics of Models 2, 3, 4 and 5 is reported in Table S5 in the Supplementary Data. For Models 3, 4 and 5 a comparison of their individual covariate importance is reported in Supplementary Table S6 and a comparison of covariate effect estimate size and odds ratios are reported in Table S7. A comparison of discrimination and calibration curves for models 2–5 is presented in Supplementary Fig. S2 and precision-recall curves in Supplementary Figure S3.

**Table 3:** Covariates included in model 2

Variable	Estimate	SE	OR	Importance	SD
Age	−0.027	0.041	0.974	0.010	0.007
APACHE III ***	0.054	0.031	1.056	0.124	0.015
Acute renal failure	−0.049	0.472	0.952	0.000	0.001
Body Mass Index	−0.078	0.104	0.925	0.012	0.009
Chronic heart disease	0.072	0.482	1.074	0.000	0.001
Chronic liver disease	−0.615	0.818	0.541	0.001	0.002
Chronic renal disease	0.444	0.922	1.559	−0.002	0.001
Chronic respiratory disease	0.180	0.512	1.197	0.002	0.001
Creatinine	−0.004	0.002	0.996	0.008	0.007
Admission after elective surgery	16.81	788	> 100	0.019	0.006
Admission after medical emergency response	−0.440	0.283	0.644	0.001	0.004
Low frailty score (1–4)	−0.586	0.364	0.556	0.002	0.004
Immunodeficiency	0.531	1.117	1.701	0.002	0.001
Immunosuppression	−0.035	0.667	0.966	0.000	0.000
Invasive ventilation day 1 ICU ***	−2.028	0.399	0.132	0.065	0.014
Plasma lactate	−0.033	0.108	0.967	0.001	0.003
Log_10_: Age	0.609	1.997	1.839	−0.007	0.005
Log_10_: APACHE III	−1.130	1.736	0.323	0.006	0.007
Log_10_: BMI	2.094	3.327	8.119	0.010	0.009
Log_10_: Creatinine	0.244	0.509	1.276	−0.004	0.004
Log_10_: Lactate level	0.534	0.393	1.705	0.001	0.007
Log_10_: P: F ratio	1.533	1.307	4.632	0.027	0.008
Log_10_: Hours in hospital prior to ICU admission	−0.005	0.102	0.995	−0.001	0.001
Log_10_: Plasma urea level	0.076	0.431	1.079	−0.003	0.002
P: F ratio ***	−0.010	0.006	0.990	0.067	0.013
Hours in hospital prior to ICU admission	−0.001	0.002	0.999	−0.001	0.002
Male sex	0.727	0.280	2.068	0.001	0.006
Plasma urea level	0.034	0.037	1.034	0.001	0.005

APACHE = (Acute Physiology and Chronic Health Evaluation), P:F Ratio = Ratio of arterial oxygen tension (mmHg) to fraction of inspired oxygen (as a decimal). All biochemical values represent the worst result reported in the first 24&nbsp;h of ICU admission. *** Represents the most influential covariates, which were selected to develop Model 5

**Fig. 3:**
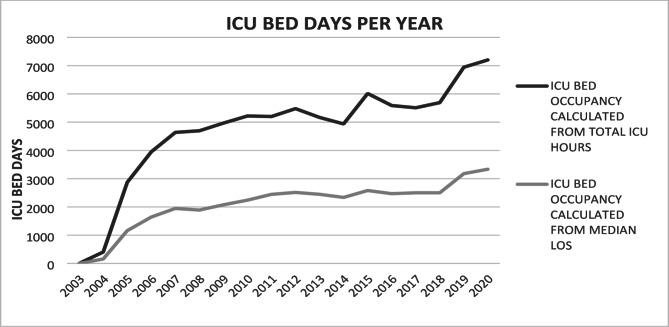
ICU bed days per year due to SAP in ANZ ICUs.Dark line: ICU bed days calculated from total number of hours for SAP patients in ICU per year, divided by 24. Light line: ICU bed days calculated by median length of stay for the designated year multiplied by the number of admissions for that year

### Burden of disease in ICUs due to AP

There was a progressive increase in annual admissions with AP to ICU with minimal change in the median LOS or proportion of patients with a LOS > 7 days (Table S7 Supplementary Appendix). Quantifying bed occupancy using median LOS values indicated 157 ICU bed days in 2004 vs. 3335 in 2020 (2003 excluded due to only 3 patient admissions in that year). When analysing total ICU bed days per year, the numbers were substantially higher; 404 in 2004 vs. 7204 in 2020 (Fig. 3).

Analysing all ICU admissions across the study period in the ANZICS-CORE database (*n* = 2,347,888), 217,605 (9·1%) had an ICU LOS > 7 days. In the medical (*n* = 1,125,701) and emergency-surgery (*n* = 341,504) subgroups, 148,948 (13.2%) and 41,456 (12%) respectively had an ICU LOS > 7 days. )

## Discussion

More than 25% of critically unwell patients in ICU with AP have a high-risk trajectory. The majority of these were attributed to a prolonged ICU LOS, with associated increases in mortality and resource use. Survivors of a prolonged ICU admission have a longer hospitalisation and are more likely to be discharged to rehabilitation or a nursing home. We found a high predictive accuracy, specificity and AUROC for our high-risk trajectory prediction model, though it was limited by low sensitivity. In our model, the greatest outcome predictors were the APACHE III score, the P:F ratio and requirement for mechanical ventilation within the first 24 h of ICU. We also observed an increasing ICU resource burden from AP as evidenced by rising admission numbers and increases in ICU bed occupancy.

To our knowledge, this is the largest study to report ICU LOS in critically unwell adults with AP, representing prospectively-collected data spanning more than 2·5 million adult intensive care admission episodes across an 18-year period. We applied robust methodology using 10-fold cross validation of the model, and trialled models at different thresholds for missing data. We employed sensitivity analyses in our model development, and minimised the competing risk of death and prolonged LOS by analysing baseline characteristics across three different cohorts. ICU LOS is a patient and institution-centred outcome, and it is reproducible and easy-to-measure in future studies. Modelling ICU LOS as a continuous variable has been challenging in prior studies due to methodological challenges [14] and we addressed this by dichotomising the LOS outcome.

This study supports previous findings that patients with critically unwell patients with AP are at risk of more complicated hospitalisation, with higher rates of mortality and prolonged ICU and hospital LOS [15]. This has implications for longer-term patient sequelae of prolonged critical illness and hospitalisation, and significant institutional ramifications with respect to resource use. The increasing number of ICU admissions coupled with a high risk of adverse patient-centred outcomes, highlights an unmet need in this patient cohort.

To improve outcomes in this patient cohort, it is necessary to risk-stratify both pancreas-specific and prolonged critical illness-related complications. We have developed a model to address this issue by predicting a composite severity outcome of mortality and prolonged ICU LOS. In our model, the strongest predictors of a high-risk trajectory were high admission illness severity and acute lung injury. Our model facilitates accurate stratification of the low-risk cohort, which could enable earlier recognition of patients suitable for a lower-intensity of care; this could be particularly useful in resource-poor settings. However, the low sensitivity of the model increases the risk that high-risk patients may be under-recognised early in the ICU admission. This is a similar limitation as other severity scoring systems for AP [16], however as shown in the Fig. 2A, different prediction thresholds could be chosen to balance sensitivity and specificity differently. Improving the sensitivity of the model may permit greater recognition of a high-risk cohort of patients. This could facilitate more targeted recruitment into future clinical trials for novel interventions in AP (rather than including patients with a low-risk trajectory). Improving prognostic enrichment in this patient cohort could then lead to more targeted management in high-risk patients. This could include more proactive establishment of enteral nutrition, earlier tracheostomisation and mobilisation to mitigate deconditioning, more targeted timing of pancreas-specific interventions, and improved resource planning within the hospital. Model 5, which only used the three most influential covariates, had nearly identical performance metrics to Model 2. Given the simplicity of the data required to produce model 5, it could have application in a wider range of settings (including resource-limited centres) and warrants exploration in future studies.

There are limitations to this study. This is a retrospective analysis and therefore the findings cannot demonstrate causality. The data is limited by the set variables collected by the ANZICS-APD. Pancreatic necrosis, a key contributor to AP outcomes, is not reported as part of the dataset. A formal a priori sample size calculation was not performed as we were limited by the number of datapoints. Based on the well-validated sample size calculation framework proposed by Riley et al. [17], our model may be underpowered, which may increase the risk of overfitting or prediction bias. However, Model 5 used only 3 covariates and had a substantially larger sample size with nearly identical performance metrics, suggesting that Model 2 is likely not overly sensitive to sample size. A priori sample size calculation should inform future studies furthering the model’s development or validation. Although cross-validation was used to internally validate the model and mitigate overfitting, external validation would be an important next step to further assess the model’s generalisability. Alternative modelling approaches using restricted cubic splines may better account for non-linear covariate relationships and would be another area for future research. More complex modelling could also account for cluster effects, and should be another focus for future research. However, our current approach provides a balance between non-linear modelling flexibility, and reproducibility of the findings as logarithmic transformations are readily implementable. While the APACHE III system is widely used in ICUs internationally, inclusion of this into the model does limit its use to systems incorporating APACHE III. The ANZICS-CORE dataset does not enable classification according to the Atlanta system, and while most cases are likely severe, the low cohort mortality in our dataset suggests that a proportion of patients have only moderately-severe AP. Finally, the dataset represents outcomes for only Australian and New Zealand ICU patients and there are likely to be differences in ICU resource availability and admission thresholds in different countries. Therefore, the model will need to be tested in other cohorts to assess the generalisability of our findings. Long-term patient-centred outcomes, such as hospital readmission or 6 or 12-month mortality, are not available with this dataset but should be a focus for exploration in future studies.

Given the low sensitivity of our prediction model (which rely on routine laboratory tests), the addition of novel biomarkers may improve outcome stratification in critically unwell patients with AP. Several immune markers have been implicated in the pathogenesis of more severe AP [18], with experimental data suggesting they may have improved prediction accuracy over conventional scoring systems [19]. Further research is required into the role of immune biomarkers and their potential role in accurately stratifying a patient’s trajectory in this patient population.

## Conclusion

A prolonged ICU admission in patients with acute pancreatitis is associated with increased morbidity, mortality and healthcare resource use. A prediction model using routinely collected data at ICU admission may enable early identification of high-risk patients, but is limited by low sensitivity. Our model, if confirmed in future studies, may present an opportunity for prognostic enrichment in patients with more severe disease.

## Supplementary Information

Supplementary Material 1.

## Data Availability

The dataset is the property of ANZICS-CORE and contributing ICUs, and is not in the public domain. Permission to review the data will need to be granted by ANZICS-CORE.

## References

[1] Barreto SG, Kaambwa B, Venkatesh K, Sasson SC, Andersen C, Delaney A, et al. Mortality and costs related to severe acute pancreatitis in the intensive care units of Australia and New Zealand (ANZ), 2003–2020. Pancreatology. 2023;23(4):341–9. 37121877 10.1016/j.pan.2023.04.006

[2] Laupland KB, Kirkpatrick AW, Kortbeek JB, Zuege DJ. Long-term mortality outcome associated with prolonged admission to the ICU. Chest. 2006;129(4):954–9. 16608944 10.1378/chest.129.4.954

[3] Johns RH, Dawson D, Ball J. Considerations and proposals for the management of patients after prolonged intensive care unit admission. Postgrad Med J. 2010;86(1019):541–51. 20702433 10.1136/pgmj.2010.100206

[4] Mederos MA, Reber HA, Girgis MD. Acute pancreatitis: a review. JAMA. 2021;325(4):382–90. 33496779 10.1001/jama.2020.20317

[5] Adult Patient Database. Australian & New Zealand Intensive Care Society (ANZICS) Centre for Outcomes & Resource Evaluation (CORE).

[6] von Elm E, Altman DG, Egger M, Pocock SJ, Gøtzsche PC, Vandenbroucke JP. The strengthening the reporting of observational studies in epidemiology (STROBE) statement: guidelines for reporting observational studies. Lancet. 2007;370(9596):1453–7. 18064739 10.1016/S0140-6736(07)61602-X

[7] Collins GS, Moons KGM, Dhiman P, Riley RD, Beam AL, Van Calster B, et al. TRIPOD + AI statement: updated guidance for reporting clinical prediction models that use regression or machine learning methods. BMJ. 2024;385: e078378. 38626948 10.1136/bmj-2023-078378 PMC11019967

[8] Lefering R, Waydhas C. Prediction of prolonged length of stay on the intensive care unit in severely injured patients-a registry-based multivariable analysis. Front Med (Lausanne). 2024;11: 1358205. 38903820 10.3389/fmed.2024.1358205 PMC11188296

[9] Wozniak H, Beckmann TS, Dos Santos Rocha A, Pugin J, Heidegger CP, Cereghetti S. Long-stay ICU patients with frailty: mortality and recovery outcomes at 6 months. Ann Intensive Care. 2024;14(1):31. 38401034 10.1186/s13613-024-01261-x PMC10894177

[10] Curran TF, Sunkara B, Leis A, Lim A, Haft J, Engoren M. Outcomes after prolonged ICU stays in postoperative cardiac surgery patients. Fed Pract. 2022;39(Suppl 5):S6-Sc11. 36923547 10.12788/fp.0300 PMC10010497

[11] van Buuren S, Groothuis-Oudshoorn K. mice: Multivariate Imputation by Chained Equations in R.

[12] Kuhn M. Building Predictive Models in R Using the caret Package.

[13] Greenwell BB. Variable importance Plots—An introduction to the vip package. R J. 2020;12:343–66.

[14] Moran JL, Duke GJ, Santamaria JD, Linden A. Modelling of intensive care unit (ICU) length of stay as a quality measure: a problematic exercise. BMC Med Res Methodol. 2023;23(1):207. 37710162 10.1186/s12874-023-02028-x PMC10500937

[15] PANC Study (Pancreatitis. A National cohort Study): national cohort study examining the first 30 days from presentation of acute pancreatitis in the UK. BJS Open. 2023;7(3). https://10.1093/bjsopen/zrad008 10.1093/bjsopen/zrad008 PMC10170253 37161673

[16] Barreto SG, Rodrigues J. Comparison of APACHE II and Imrie scoring systems in predicting the severity of acute pancreatitis. World J Emerg Surg. 2007;2:33. 18067678 10.1186/1749-7922-2-33 PMC2169219

[17] Riley RD, Ensor J, Snell KIE, Harrell FE Jr., Martin GP, Reitsma JB, et al. Calculating the sample size required for developing a clinical prediction model. BMJ. 2020;368: m441. 32188600 10.1136/bmj.m441

[18] Venkatesh K, Glenn H, Delaney A, Andersen CR, Sasson SC. Fire in the belly: a scoping review of the immunopathological mechanisms of acute pancreatitis. Front Immunol. 2022;13: 1077414. 36713404 10.3389/fimmu.2022.1077414 PMC9874226

[19] Wiley MB, Mehrotra K, Bauer J, Yazici C, Bialkowska AB, Jung B. Acute pancreatitis: current clinical approaches, molecular pathophysiology, and potential therapeutics. Pancreas. 2023;52(6):e335-43. 38127317 10.1097/MPA.0000000000002259 PMC11913250

