# Supplementary material for: A prediction model for ‘ICU mortality or prolonged ICU stay’ in critically unwell patients with acute pancreatitis: insights from a 2003–2020 cohort analysis using the ANZICS-CORE database
Source: Crit Care. 2025 Aug 6;29:347. doi: 10.1186/s13054-025-05590-6 (PMC12326670; doi:10.1186/s13054-025-05590-6)
Supplement: Supplementary file 1 — Supplementary Material 1. [file 13054_2025_5590_MOESM1_ESM.docx]

**Supplementary Appendix**

| **Data Variable** | **Description** |
| --- | --- |
| Indigenous origin | Indicates Indigenous status of a patient |
| ECMO Indicator | Indicates delivery of ECMO during patient’s ICU stay |
| GCS unavailable due to sedation | Indicates GCS components not available due to sedation |
| ICU admission following elective surgery | An ICU admission directly following an elective surgery |
| Inotropes/vasopressor indicator | Indicates administration of inotropes or vasopressors during the patient’s stay in ICU |
| Invasive Ventilation Status for Respiratory rate (Low) | Indicates invasive ventilation status of a patient at the time of the lowest respiratory rate during their first 24hrs in ICU |
| Invasive ventilation status for respiratory rate (High) | Indicates invasive ventilation status of a patient at the time of the highest respiratory rate during their first 24hrs in ICU |
| Invasive ventilation indicator | Indicates delivery of invasive ventilation during the patient’s stay in ICU |
| Invasively ventilated on Day 1 | Identifies whether a patient received invasive ventilation during their first 24 hours in ICU |
| Non-invasive ventilation indicator | Indicates delivery of non-invasive ventilation during the patient’s stay in ICU |
| Planned ICU admission | A planned admission to ICU |
| Renal replacement therapy indicator | Indicator of renal replacement therapy during ICU stay |
| Statistical linkage key | To enable data linkage while maintaining patient privacy |
| Tracheostomy indicator | Indicates tracheostomy performed during the patient’s current stay in ICU |
| Diabetes status | Diabetes status of a patient at time of hospital admission |
| Clinical frailty score | Patient’s frailty assessment at time of Hospital admission |
| Invasive ventilation hours | Total invasive ventilation hours during patient’s stay in ICU |
| Lactate | Highest lactate value (first 24 hours) |
| Non-invasive ventilation hours | Total non-invasive ventilation hours during patient’s stay in ICU |
| Delirium | An indicator of whether the patient developed delirium during the current ICU episode |
| Pressure injury | An indicator of whether the patient developed a pressure injury during the current ICU episode |

**Table S1:** Dataset variables added to the ANZICS-CORE Adult Patient Database in 2016

| **Variable** | **Rationale for inclusion** |
| --- | --- |
| ***Demographic*** | |
| Age | Standard covariate in critical illness prediction scores and an independent predictor of ICU mortality and poorer outcomes. (1-3) |
| Sex | Exploratory analysis to assess impact of sex on outcomes in critically unwell AP patients; there are known sex-based outcome differences in different critically unwell cohorts. (4, 5) |
| Chronic Respiratory failure | Clinically justifiable comorbidity that could worsen outcomes in critically unwell AP patients, particularly given the risk of acute respiratory failure in this cohort. |
| Chronic cardiovascular failure | Clinically justifiable comorbidity that worsens outcomes in critically unwell patients. |
| End-stage renal failure | Clinically justifiable comorbidity that could worsen outcomes in critically unwell AP patients , particularly given the risk of acute kidney injury in this cohort. |
| Chronic liver disease | Clinically justifiable comorbidity that worsens outcomes in critically unwell patients. |
| Immunodeficiency | Clinically justifiable comorbidity that worsens outcomes in critically unwell patients. |
| Immunosuppressive therapy | Clinically justifiable comorbidity that worsens outcomes in critically unwell patients. |
| Frailty score (1-4, low risk vs. 5-8, high risk) | Frailty associated with poorer outcomes in critically unwell patients in ICU, therefore may represent a prognostic comorbidity marker in AP patients. (6) |
| Body mass index | Risk factor for mortality and poorer outcomes in critically unwell cohorts (7, 8) and would be expected to worsen outcome in AP. |
| ***Biochemical*** | |
| Urea | Reproducible test. Independently associated with severity and is a covariate in various AP severity scoring systems (9). |
| Creatinine | Reproducible test associated with acute kidney injury and critical illness. |
| Lactate | Well-validated marker of illness severity and mortality risk in critically unwell patients. (10, 11) |
| ***Clinical/Physiological*** | |
| Admission following elective surgery | AP admissions after elective surgery would be expected to have a better clinical outcome. |
| Invasive ventilation day 1 ICU | Clinically justifiable covariate, as both a marker of illness severity and likely respiratory failure. |
| Hours in hospital prior to ICU admission | Clinical marker of rapidity of disease progression, to assess whether patients who have a delay to ICU admission are at risk of poorer outcome. |
| Admission following emergency response call | Marker of illness severity in patients admitted to intensive care with associated poorer outcomes (12). In patients with AP, would represent a marker of acute deterioration. |
| Acute renal failure | Marker of illness severity in both critically unwell patients and with AP. |
| APACHE III score | Well-validated illness severity score for critically unwell patients in ICU. |
| PaO2:FiO2 ratio | Well-validated, reproducible marker of acute lung injury. Clinically justifiable covariate given the high risk of acute respiratory failure in critically unwell AP patients. |

**Table S2:** Variables selected for inclusion into LOS prediction model. All datapoints could only include data available within the first 24 hours of ICU admission.

MET = Medical Emergency team

APACHE III = Acute Physiology and Chronic Health Evaluation III score

P:F ratio = Ratio of arterial oxygen tension (mmHg) to fraction of inspired oxygen (%

|  | Total n = 13275 | LOS ≤7 days  n = 10415 (78.4%) | LOS >7 days  n = 2860 (21.6%) | P value* |
| --- | --- | --- | --- | --- |
| Mean age (SD) | 59 (18) | 59 (18) | 59 (17) | 0.11 |
| Mean APACHE III (SD) | 56 (26) | 52 (25) | 69 (25) | <0.001 |
| Male sex, n (%) | 7911 (60) | 6033 (58) | 1878 (66) | <0.001 |
| Admission due to emergency response call, n (%) | 1941 (18) | 1424 (16) | 517 (22) | <0.001 |
|  |  |  |  |  |
| Clinical status within first 24 hours of ICU admission | | | | |
| *Invasive ventilation, n (%)* | 557 (15) | 281 (9) | 276 (38) | <0.001 |
| *Acute kidney injury, n (%)* | 1420 (11) | 863 (8) | 557 (20) | <0.001 |
| *Urea, mean (SD)* | 9.4 (8.3) | 8.8 (8) | 11.8 (8.5) | <0.001 |
| *Lactate, mean (SD)* | 2.6 (2.8) | 2.5 (2.8) | 3 (2.6) | <0.001 |
| *P:F ratio, mean (SD)* | 255 (137) | 273 (137) | 204 (122) | <0.001 |
|  |  |  |  |  |
| Interventions received during ICU admission | | | | |
| *Invasive ventilation^a^* | 726 (23) | 315 (13) | 411 (64) | <0.001 |
| *Inotropes^a^* | 917 (32) | 571 (25) | 346 (60) | <0.001 |
| *Renal replacement therapy^a^* | 377 (13) | 153 (7) | 224 (39) | <0.001 |
|  |  |  |  |  |
| Chronic conditions |  |  |  |  |
| *Chronic respiratory* | 596 (4.5) | 482 (4.6) | 114 (3.9) | 0.14 |
| *Chronic cardiovascular* | 944 (7.1) | 763 (7.3) | 181 (6.3) | 0.07 |
| *Chronic liver* | 312 (2.4) | 244 (2.3) | 68 (2.4) | 0.82 |
| *Chronic renal* | 368 (2.8) | 279 (2.7) | 89 (3.1) | 0.21 |
| *Immunosuppressive treatment* | 337 (2.5) | 280 (2.7) | 57 (2.0) | 0.05 |
|  |  |  |  |  |
| Outcomes |  |  |  |  |
| ICU mortality, n (%) | 1022 (7.7) | 693 (6.6) | 329 (11.6) | <0.001 |
| Hospital mortality, n( %) | 1480 (11.2) | 983 (9.4) | 497 (17.5) | <0.001 |
| Hospital LOS (hours), median (IQR 1-3) | 273 (148-512) | 218 (123-372) | 642 (402-1196) | <0.001 |
|  |  |  |  |  |
| *Hospital discharge destination (as a proportion of hospital survivors only)* | | | | |
| Home, n (%) | 9114 (80) | 7554 (78) | 1560 (67) | <0.001 |
| Rehab/other hospital, n (%) | 2062 (18) | 1498 (16) | 564 (24) | <0.001 |
| Nursing home, n (%) | 497 (4) | 297 (3) | 200 (9) | <0.001 |

**Table S3:** Baseline characteristics comparing patients with a LOS ≤7 days vs. >7 days (irrespective of survival status).

APACHE = (Acute Physiology and Chronic Health Evaluation)

^a^These variables were only available from 2017 onwards. Data on mechanical ventilation (n = 3102), inotropes (n = 2902), renal replacement therapy (n = 2811)

|  | Total n = 13275 | Survived ICU n = 12253 (92.3%) | Died ICU n = 1022 (7.7%) | P value* |
| --- | --- | --- | --- | --- |
| Mean age (SD) | 59 (18) | 58 (18) | 70 (14) | <0.001 |
| Mean APACHE III (SD) | 56 (26) | 52 (23) | 99 (30) | <0.001 |
| Male sex, n (%) | 7911 (60) | 7292 (60) | 619 (61) | 0.49 |
| Admission due to emergency response call, n (%) | 1941 (18) | 1723 (17) | 218 (27) | <0.001 |
|  |  |  |  |  |
| Clinical status within first 24 hours of ICU admission | | | | |
| *Invasive ventilation, n (%)* | 557 (15) | 406 (12) | 151 (53) | <0.001 |
| *Acute kidney injury, n (%)* | 1420 (11) | 959 (8) | 461 (46) | <0.001 |
| *Urea, mean (SD)* | 9.4 (8.3) | 8.9 (8.0) | 15.9 (8.2) | <0.001 |
| *Lactate, mean (SD)* | 2.6 (2.8) | 2.3 (2.2) | 6.5 (4.9) | <0.001 |
| *P:F ratio, mean (SD)* | 255 (137) | 263 (137) | 182 (114) | <0.001 |
|  |  |  |  |  |
| Interventions received during ICU admission | | | | |
| *Invasive ventilation^a^* | 726 (23) | 543 (19) | 183 (75) | <0.001 |
| *Inotropes^a^* | 917 (32) | 725 (27) | 192 (84) | <0.001 |
| *Renal replacement therapy^a^* | 377 (13) | 238 (9) | 139 (60) | <0.001 |
|  |  |  |  |  |
| Chronic conditions |  |  |  |  |
| *Chronic respiratory* | 596 (4.5) | 537 (4.4) | 59 (5.8) | 0.03 |
| *Chronic cardiovascular* | 944 (7.1) | 858 (7.0) | 86 (8.4) | 0.08 |
| *Chronic liver* | 312 (2.4) | 279 (2.3) | 33 (3.2) | 0.05 |
| *Chronic renal* | 368 (2.8) | 322 (2.6) | 46 (4.5) | <0.001 |
| *Immunosuppressive treatment* | 337 (2.5) | 310 (2.5) | 27 (2.6) | 0.83 |
|  |  |  |  |  |
| Outcomes |  |  |  |  |
| ICU LOS, median (IQR 1-3) | 67 (33-144) | 68 (34-141) | 66 (29-257) | N.A |
| ICU LOS, mean (SD) | 152 (290) | 146 (277) | 235 (418) | 0.03 |

**Table S4:** Baseline characteristics and outcomes comparing patients who survived ICU vs. died in ICU

APACHE = (Acute Physiology and Chronic Health Evaluation)

^a^These variables were only available from 2017 onwards. Data on mechanical ventilation (n = 3102), inotropes (n = 2902), renal replacement therapy (n = 2811)

| **Metric** | **Model 2** | **Model 3** | **Model 4** | **Model 5** |
| --- | --- | --- | --- | --- |
| **LOS threshold** | 7 days | 5 days | 4 days | 7 days |
| **Covariates included** | All | All | All | APACHE III  Day 1 ventilation  PaO2:FiO2 ratio |
| **Sample size** | 542 | 542 | 542 | 2264 |
| **High-risk outcome rate** | 174 (32.1%) | 235 (43.4%) | 267 (49.3%) | 675 (29.8%) |
| **Expected accuracy** | 59.6 | 52 | 50.1 | 61.9 |
| **Accuracy** | 79.5 | 73.8 | 71.2 | 80.2 |
| **Kappa** | 0.493 | 0.455 | 0.423 | 0.479 |
| **Sensitivity (high-risk)** | 54 | 60.4 | 64.8 | 51.1 |
| **Specificity (high-risk)** | 91.6 | 84 | 77.5 | 92.5 |
| **AUROC** | 82.9 | 80.2 | 78.8 | 83 |
| **Precision (low-risk)** | 80.8 | 73.5 | 69.4 | 81.7 |
| **Precision (high-risk)** | 75.2 | 74.3 | 73.6 | 74.4 |
| **AUC Precision (low-risk)** | 90.5 | 82.8 | 77.2 | 90.5 |
| **AUC Precision (high-risk)** | 71.8 | 76.8 | 78.1 | 71 |
| **F1 (low-risk)** | 85.9 | 78.4 | 73.2 | 86.8 |
| **F1 (high-risk)** | 62.9 | 66.7 | 68.9 | 60.6 |
| **Brier** | 0.148 | 0.178 | 0.189 | 0.143 |

**Table S5:** Prediction model metrics comparing Models 2, 3, 4, 5

|  | **Model 3** | | **Model 4** | | **Model 5** | |
| --- | --- | --- | --- | --- | --- | --- |
| **Variable** | Importance | SD | Importance | SD | Importance | SD |
| **APACHE III** | 0.133 | 0.014 | 0.112 | 0.016 | 0.085 | 0.006 |
| **PaO2:FiO2** | 0.047 | 0.012 | 0.084 | 0.015 | 0.040 | 0.006 |
| **Invasive ventilation day 1** | 0.054 | 0.012 | 0.041 | 0.010 | 0.045 | 0.005 |
| **Log_10_ PaO2:FiO2** | 0.017 | 0.007 | 0.027 | 0.011 | 0.011 | 0.003 |
| **Body mass index** | 0.035 | 0.011 | 0.053 | 0.013 |  |  |
| **Age** | 0.032 | 0.009 | 0.029 | 0.010 |  |  |
| **Log_10_ Body Mass Index** | 0.033 | 0.010 | 0.056 | 0.010 |  |  |
| **Creatinine** | 0.009 | 0.006 | 0.005 | 0.005 |  |  |
| **Log_10_ APACHE III** | 0.020 | 0.008 | 0.014 | 0.007 |  |  |
| **Frailty** | 0.001 | 0.003 | 0.000 | 0.001 |  |  |
| **Immunodeficiency** | 0.003 | 0.002 | 0.004 | 0.003 |  |  |
| **Chronic respiratory disease** | -0.001 | 0.002 | 0.013 | 0.006 |  |  |
| **Lactate** | 0.008 | 0.008 | 0.003 | 0.008 |  |  |
| **Urea** | 0.004 | 0.003 | 0.010 | 0.006 |  |  |
| **Sex** | 0.014 | 0.007 | 0.017 | 0.008 |  |  |
| **Log_10_ Lactate** | 0.053 | 0.013 | 0.050 | 0.012 |  |  |
| **Emergency response call** | 0.001 | 0.004 | 0.016 | 0.008 |  |  |
| **Chronic liver disease** | 0.000 | 0.001 | -0.002 | 0.002 |  |  |
| **Immunosuppression** | 0.000 | 0.002 | 0.000 | 0.002 |  |  |
| **Acute renal failure** | -0.001 | 0.003 | 0.000 | 0.003 |  |  |
| **Chronic cardiovascular disease** | 0.000 | 0.000 | -0.001 | 0.001 |  |  |
| **Log_10_ Hours prior to ICU** | 0.003 | 0.001 | 0.000 | 0.003 |  |  |
| **Hours prior to ICU** | 0.014 | 0.004 | -0.001 | 0.002 |  |  |
| **Chronic renal disease** | 0.000 | 0.000 | -0.002 | 0.002 |  |  |
| **Log_10_ Urea** | 0.011 | 0.008 | 0.021 | 0.012 |  |  |
| **Log_10_ Creatinine** | 0.013 | 0.010 | 0.006 | 0.008 |  |  |
| **Log_10_ Age** | 0.006 | 0.004 | -0.001 | 0.003 |  |  |

**Table S6:** Individual covariate importance and standard deviation (SD) for Models 2, 3, 4 and 5.

|  | **Model 3** | | | **Model 4** | | | **Model 5** | | |
| --- | --- | --- | --- | --- | --- | --- | --- | --- | --- |
| **Variable** | **Estimate** | **SE** | **OR** | **Estimate** | **SE** | **OR** | **Estimate** | **SE** | **OR** |
| Sex | -0.461 | 0.242 | 0.631 | -0.454 | 0.232 | 0.635 |  |  |  |
| Hours prior to ICU | -0.001 | 0.001 | 0.999 | 0.000 | 0.001 | 1.000 |  |  |  |
| Age | 0.024 | 0.036 | 1.024 | 0.022 | 0.035 | 1.022 |  |  |  |
| Chronic respiratory disease | 0.358 | 0.460 | 1.431 | 0.863 | 0.468 | 2.369 |  |  |  |
| Chronic cardiovascular disease | 0.021 | 0.437 | 1.021 | -0.095 | 0.432 | 0.909 |  |  |  |
| Chronic liver disease | 0.057 | 0.778 | 1.059 | -0.237 | 0.762 | 0.789 |  |  |  |
| Chronic renal disease | 0.026 | 0.801 | 1.026 | 0.311 | 0.794 | 1.365 |  |  |  |
| Immunodeficiency | -1.348 | 1.152 | 0.260 | -2.049 | 1.138 | 0.129 |  |  |  |
| Immunosuppression | -0.344 | 0.659 | 0.709 | 0.175 | 0.686 | 1.191 |  |  |  |
| Invasive ventilation day 1 | 1.880 | 0.426 | 6.553 | 1.674 | 0.410 | 5.331 | 1.408 | 0.134 | 4.089 |
| Emergency response call | 0.210 | 0.261 | 1.234 | 0.477 | 0.260 | 1.611 |  |  |  |
| Acute renal failure | -0.514 | 0.479 | 0.598 | -0.213 | 0.496 | 0.808 |  |  |  |
| Urea | 0.008 | 0.034 | 1.008 | 0.028 | 0.032 | 1.028 |  |  |  |
| Creatinine | 0.002 | 0.002 | 1.002 | 0.001 | 0.002 | 1.001 |  |  |  |
| Lactate | 0.110 | 0.103 | 1.117 | 0.094 | 0.104 | 1.099 |  |  |  |
| APACHE III | -0.050 | 0.029 | 0.951 | -0.042 | 0.028 | 0.959 | -0.036 | 0.003 | 0.965 |
| Body Mass Index | 0.076 | 0.086 | 1.079 | 0.090 | 0.084 | 1.094 |  |  |  |
| Frailty score | 0.197 | 0.342 | 1.217 | -0.036 | 0.341 | 0.965 |  |  |  |
| PaO2:FiO2 | 0.005 | 0.005 | 1.005 | 0.007 | 0.005 | 1.007 | 0.007 | 0.002 | 1.007 |
| Log10 Hours prior to ICU | -0.006 | 0.089 | 0.994 | -0.020 | 0.087 | 0.980 |  |  |  |
| Log10 Age | -0.185 | 1.752 | 0.831 | -0.063 | 1.695 | 0.939 |  |  |  |
| Log10 Urea | -0.246 | 0.373 | 0.782 | -0.407 | 0.355 | 0.665 |  |  |  |
| Log10 Creatinine | -0.334 | 0.446 | 0.716 | -0.188 | 0.437 | 0.828 |  |  |  |
| Log10 Lactate | -0.794 | 0.351 | 0.452 | -0.752 | 0.341 | 0.471 |  |  |  |
| Log10 APACHE III | 0.877 | 1.522 | 2.403 | 0.582 | 1.437 | 1.790 |  |  |  |
| Log10 Body Mass Index | -1.946 | 2.788 | 0.143 | -2.554 | 2.730 | 0.078 |  |  |  |
| Log10 PaO2:FiO2 | -0.506 | 1.129 | 0.603 | -0.951 | 1.096 | 0.386 | -0.613 | 0.406 | 0.542 |

**Table S7:** Models 2, 3, 4 and 5 covariate estimate effect size, standard error (SE) and Odds ratio (OR)

| Year | Number of patients | Mean age (SD) | % Male | Mean APACHE III Score (SD) | Median LOS in hours (IQR 1-3) | Number of patients with LOS > 1 week | % patients with LOS > 1 week |
| --- | --- | --- | --- | --- | --- | --- | --- |
| 2003 | 3 | 75 (9) | 0 | 48 (16) | 21 (18-29) | 0 | 0 |
| 2004 | 53 | 57 (17) | 57 | 48 (26) | 71 (26-191) | 16 | 30 |
| 2005 | 416 | 61 (17) | 59 | 56 (29) | 67 (29-159) | 100 | 24 |
| 2006 | 580 | 62 (17) | 60 | 54 (27) | 68 (34-141) | 123 | 21 |
| 2007 | 708 | 61 (18) | 60 | 56 (27) | 66 (30-150) | 162 | 23 |
| 2008 | 709 | 60 (18) | 58 | 56 (29) | 64 (28-165) | 175 | 25 |
| 2009 | 722 | 60 (18) | 59 | 58 (30) | 69 (33-159) | 168 | 23 |
| 2010 | 747 | 62 (17) | 61 | 57 (26) | 72 (33-182) | 204 | 27 |
| 2011 | 804 | 60 (18) | 57 | 58 (26) | 73 (36-164) | 195 | 24 |
| 2012 | 838 | 59 (18) | 55 | 56 (26) | 72 (37-147) | 191 | 23 |
| 2013 | 839 | 58 (18) | 58 | 56 (27) | 70 (33-146) | 189 | 23 |
| 2014 | 837 | 60 (18) | 59 | 55 (25) | 67 (36-142) | 171 | 20 |
| 2015 | 898 | 58 (19) | 58 | 56 (26) | 69 (33-154) | 201 | 22 |
| 2016 | 941 | 58 (18) | 62 | 54 (27) | 63 (29-138) | 184 | 20 |
| 2017 | 984 | 58 (18) | 60 | 54 (24) | 61 (32-124) | 178 | 18 |
| 2018 | 1000 | 59 (18) | 56 | 55 (25) | 60 (30-121) | 170 | 17 |
| 2019 | 1090 | 58 (18) | 62 | 55 (25) | 70 (37-138) | 213 | 20 |
| 2020 | 1160 | 55 (19) | 65 | 55 (25) | 69 (39-139) | 223 | 19 |

**Table S8:** Trends in demographics, illness severity and LOS for each study year.

APACHE III = Acute Physiology and Chronic Health Evaluation III score


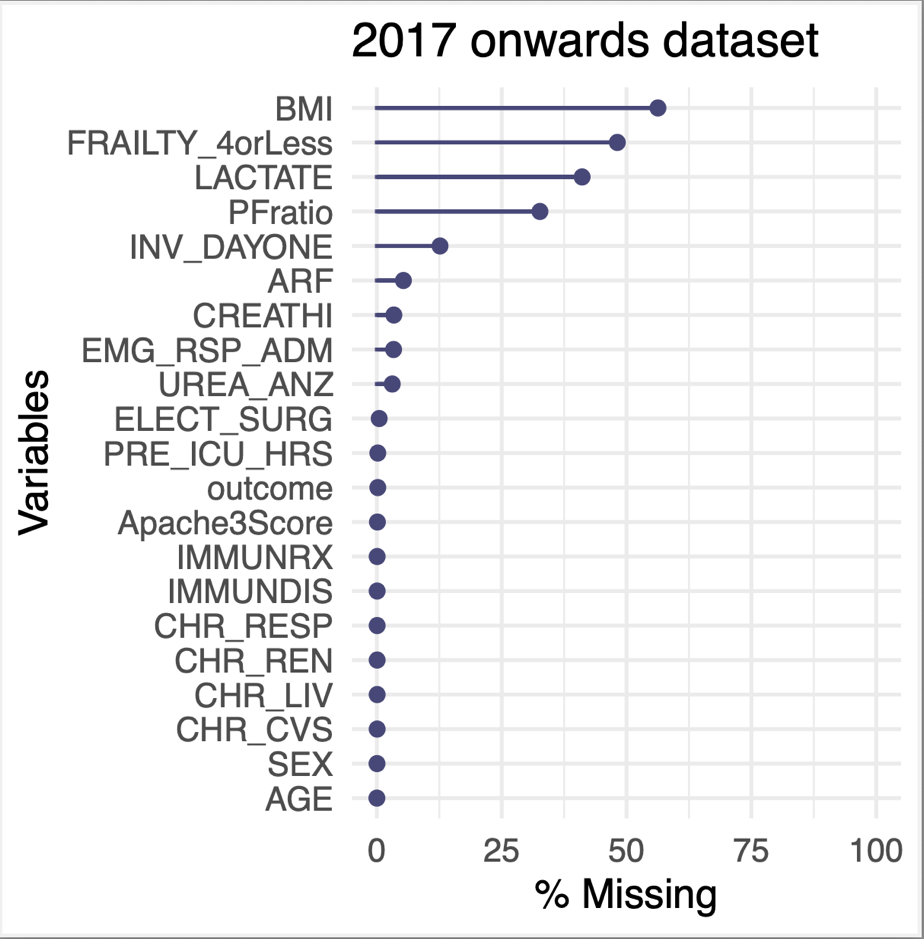

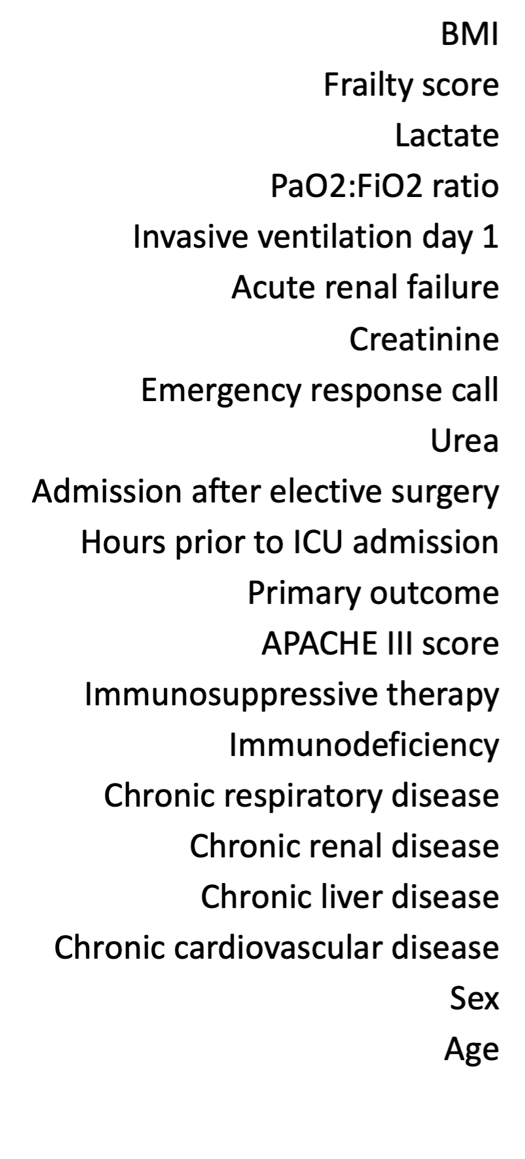


**A**

**Percentage of missing datapoints using data from 2017 onwards**

**Datapoints missing (%)**


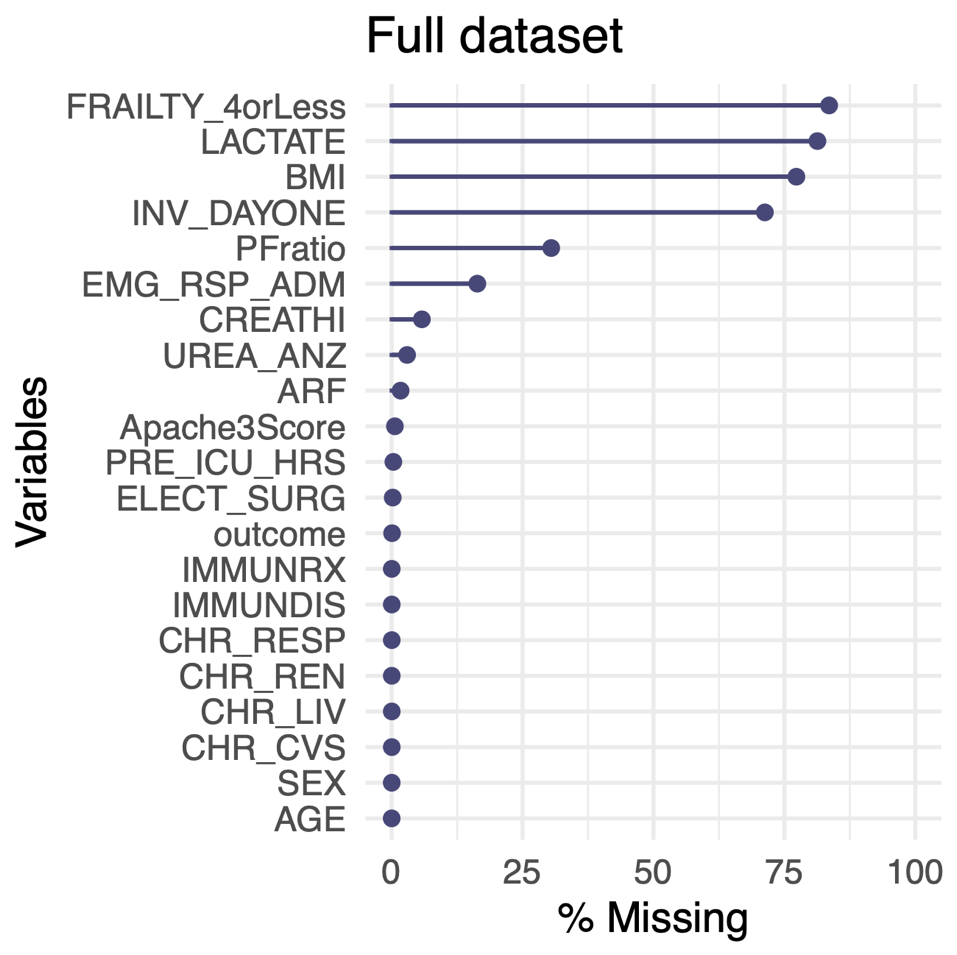

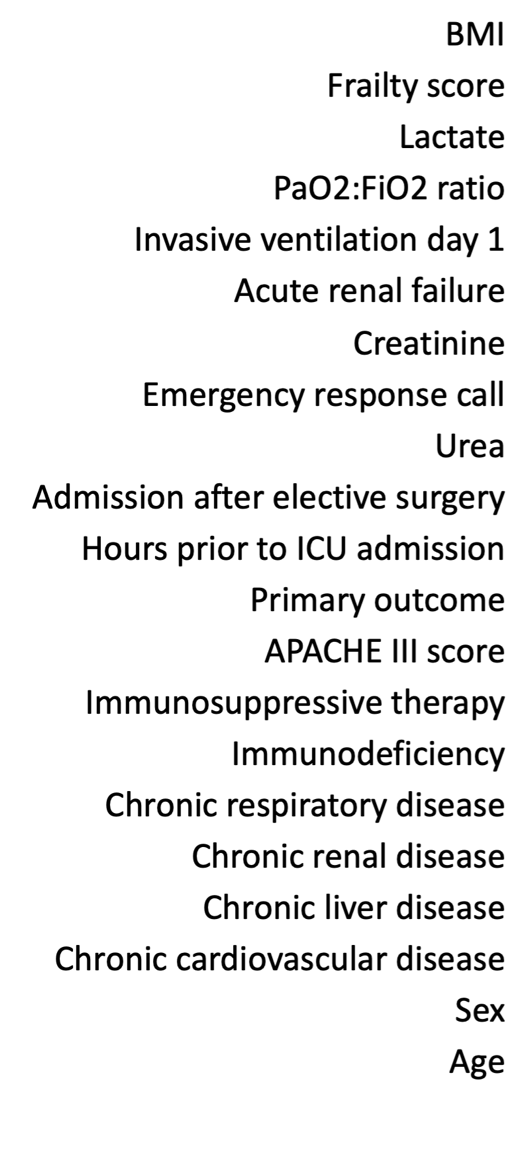


**B**

**Percentage of missing datapoints using entire dataset**

**Figure S1:** A) Percentage of missing datapoints per covariate for the dataset using only data from 2017 onwards. B) Percentage of missing datapoints per covariate across the whole dataset


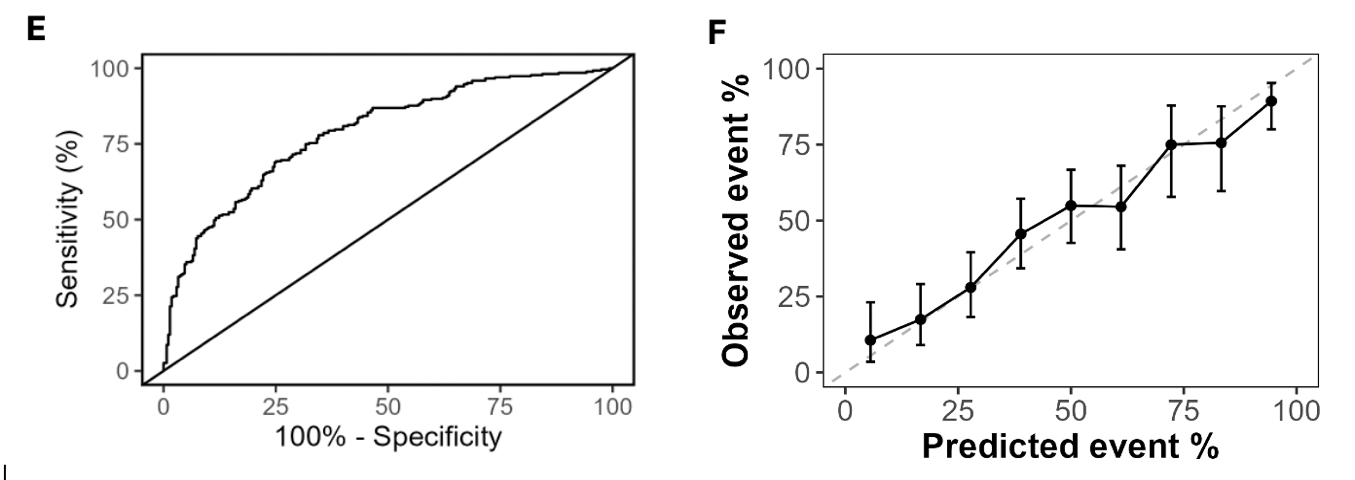

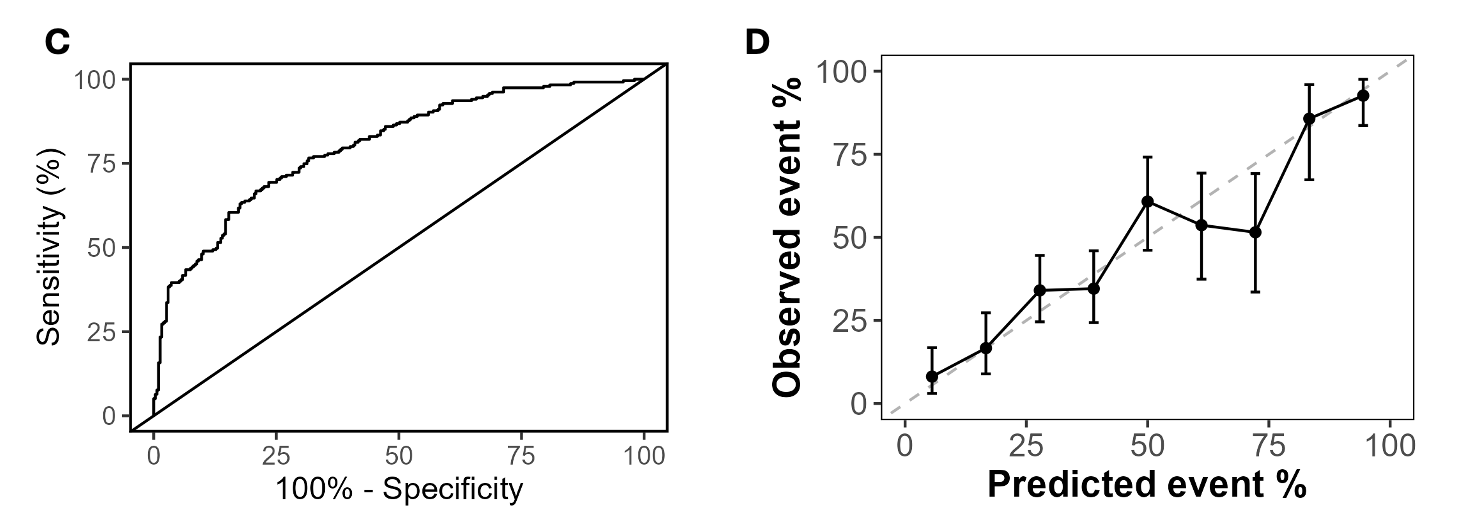

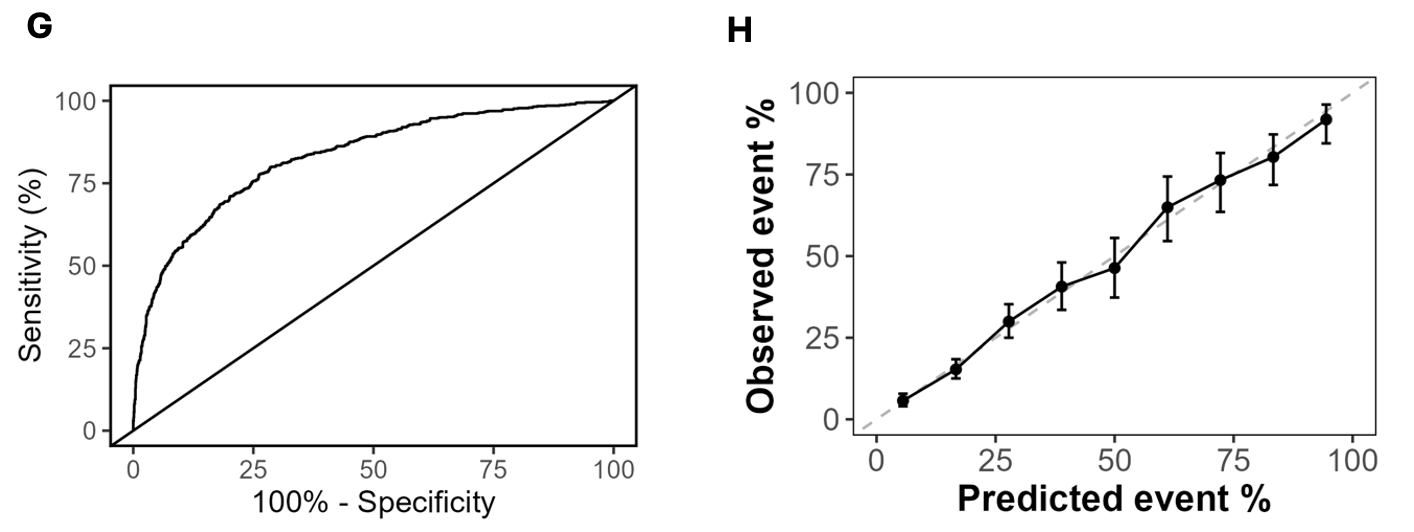

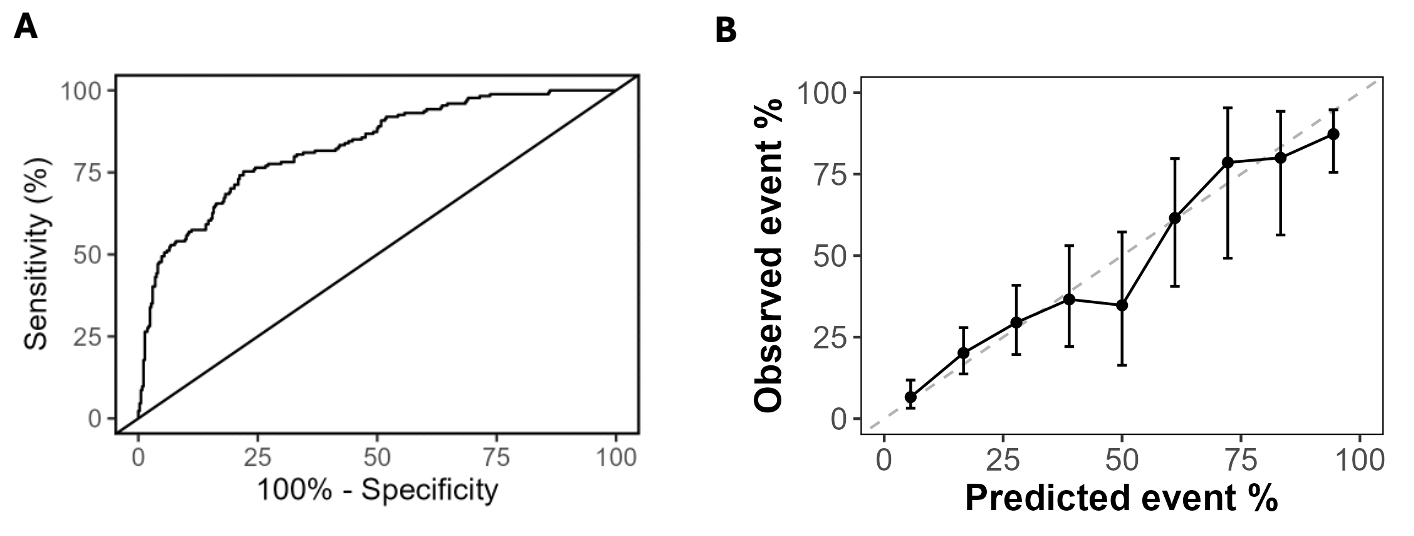


**Figure S2:** A) Model 2 Discrimination curve AUROC 0.829 B) Model 2 Calibration curve Brier 0.148 C) Model 3 Discrimination curve AUROC 0.802 D) Model 3 Calibration curve Brier 0.178 E) Model 4 Discrimination curve AUROC 0.788 F) Model 4 Calibration curve Brier 0.189 G) Model 5 Discrimination curve AUROC 0.83 H) Model 5 Calibration curve Brier 0.143


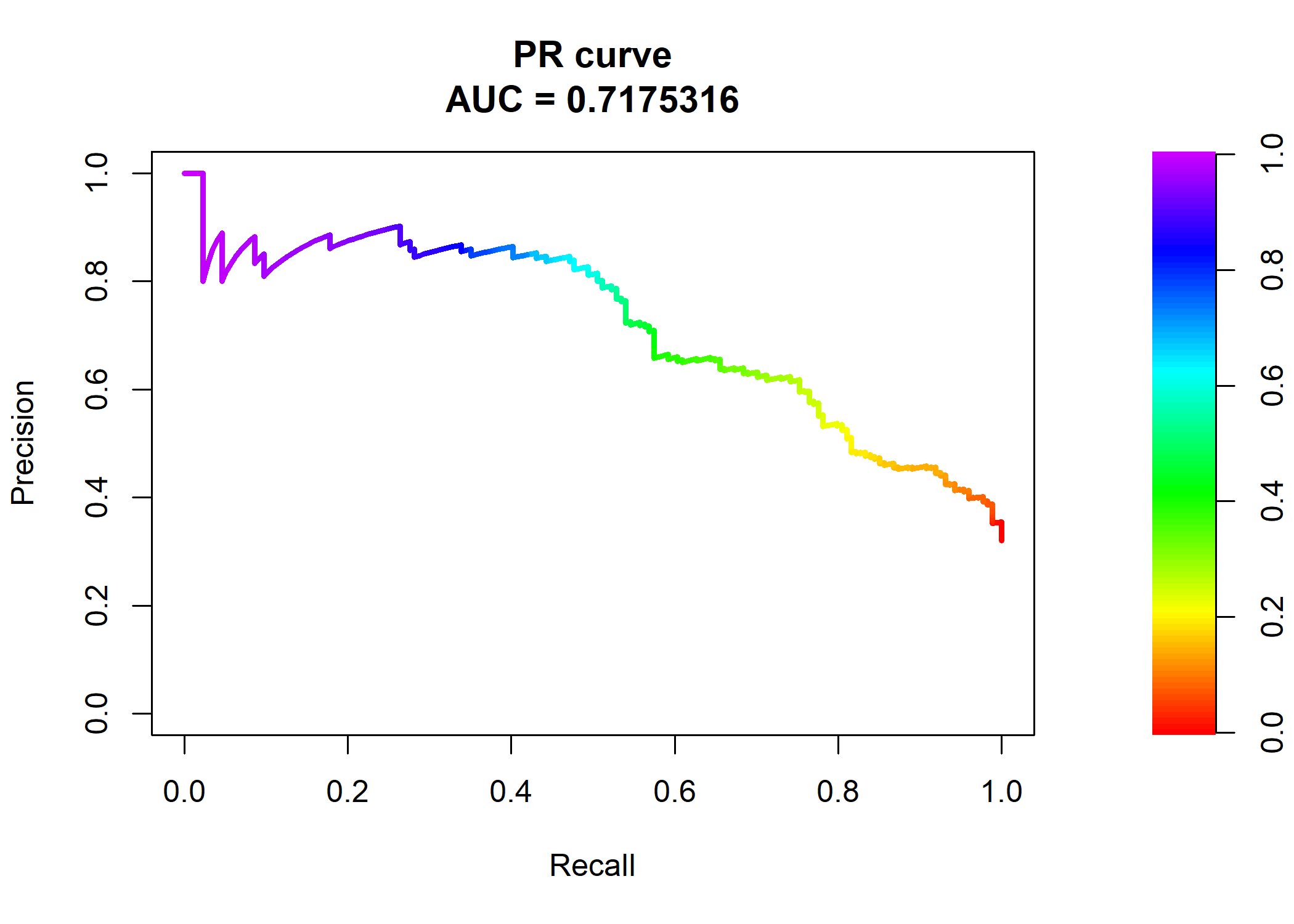

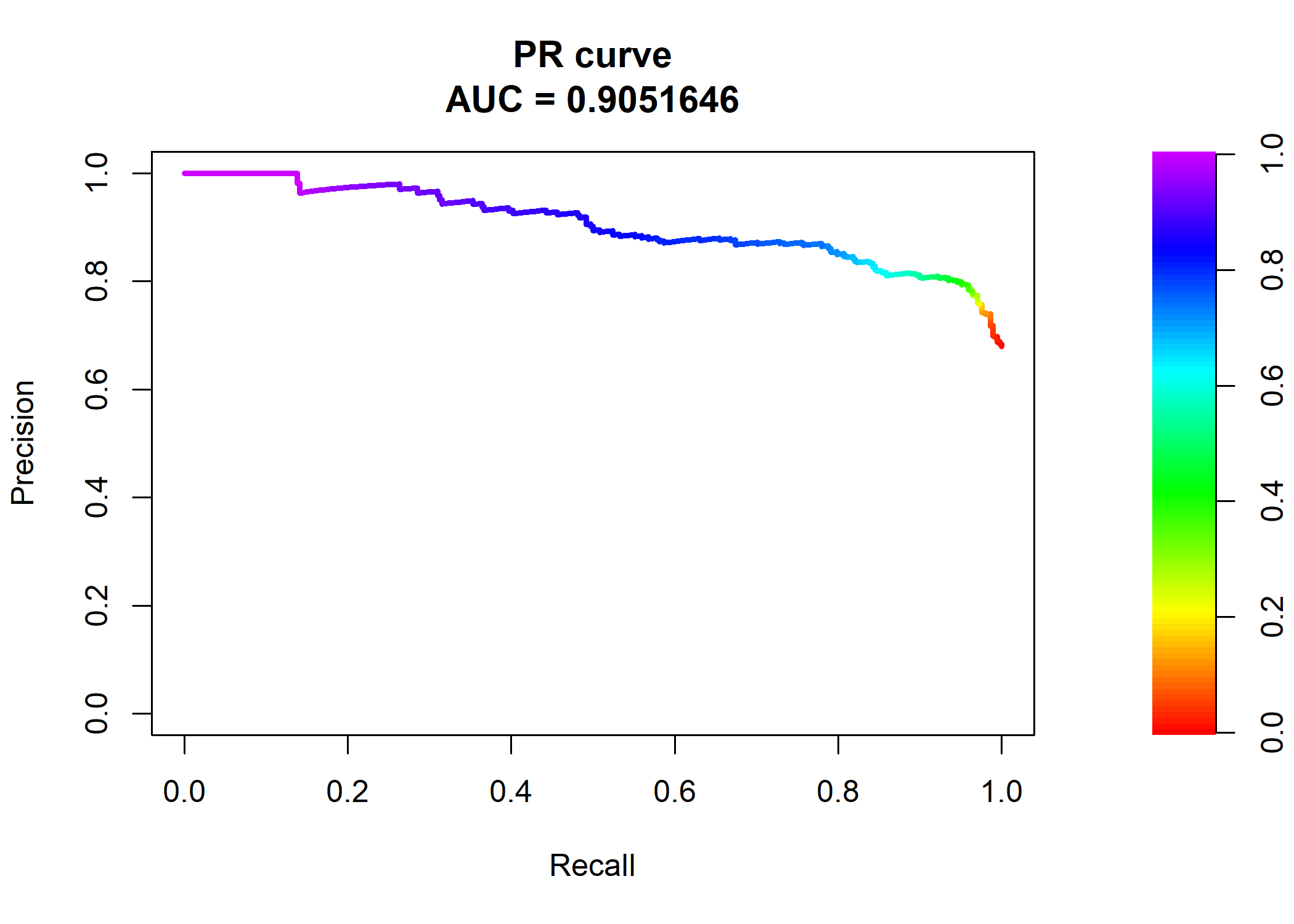


**A**

**B**


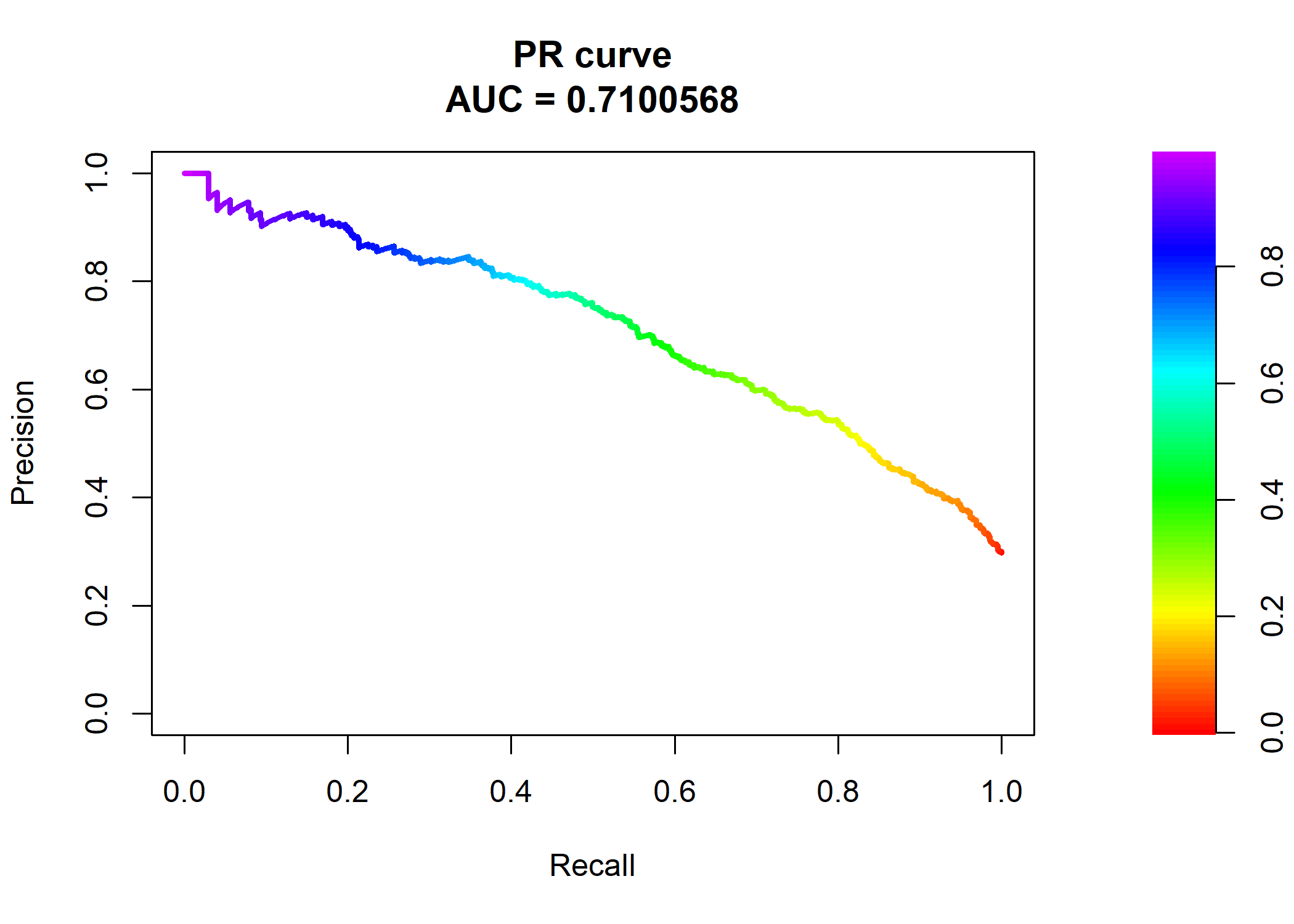


**G**


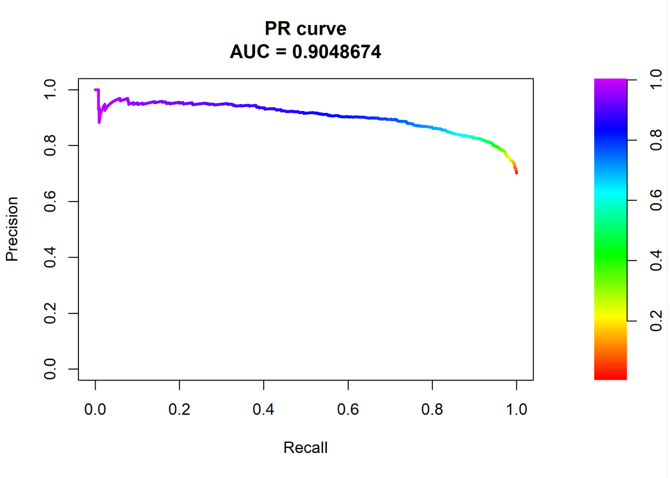


**H**


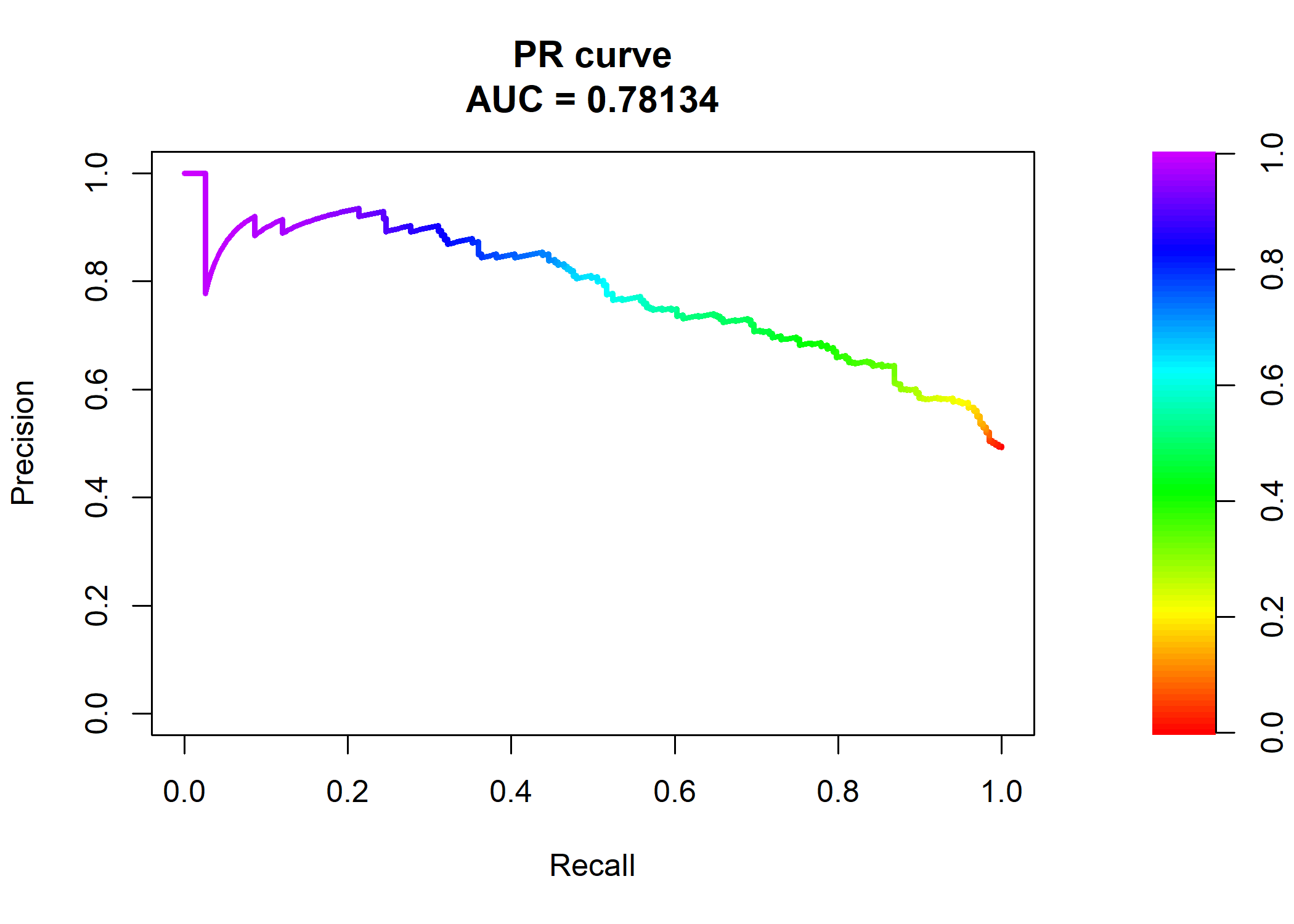

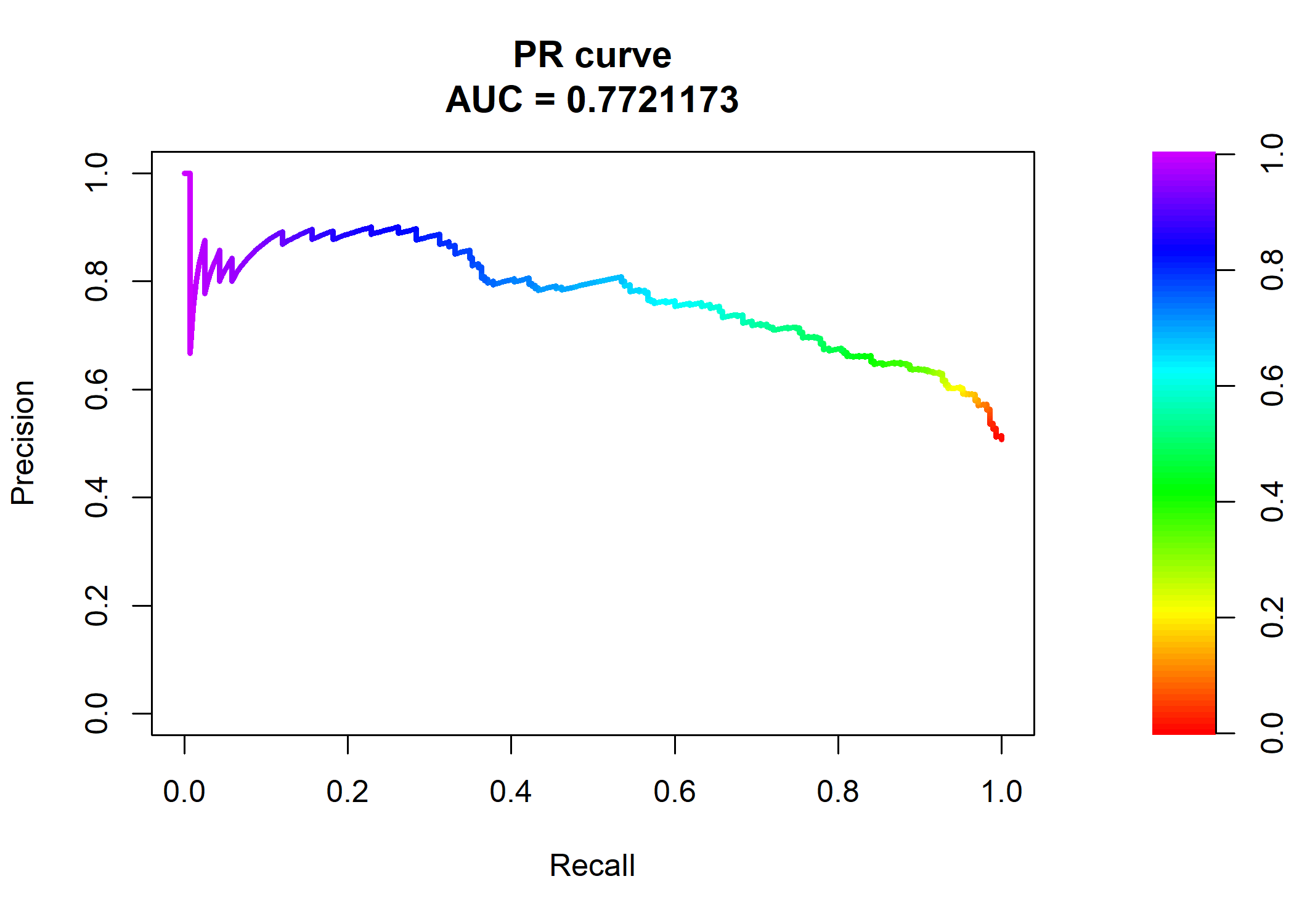


**F**

**E**


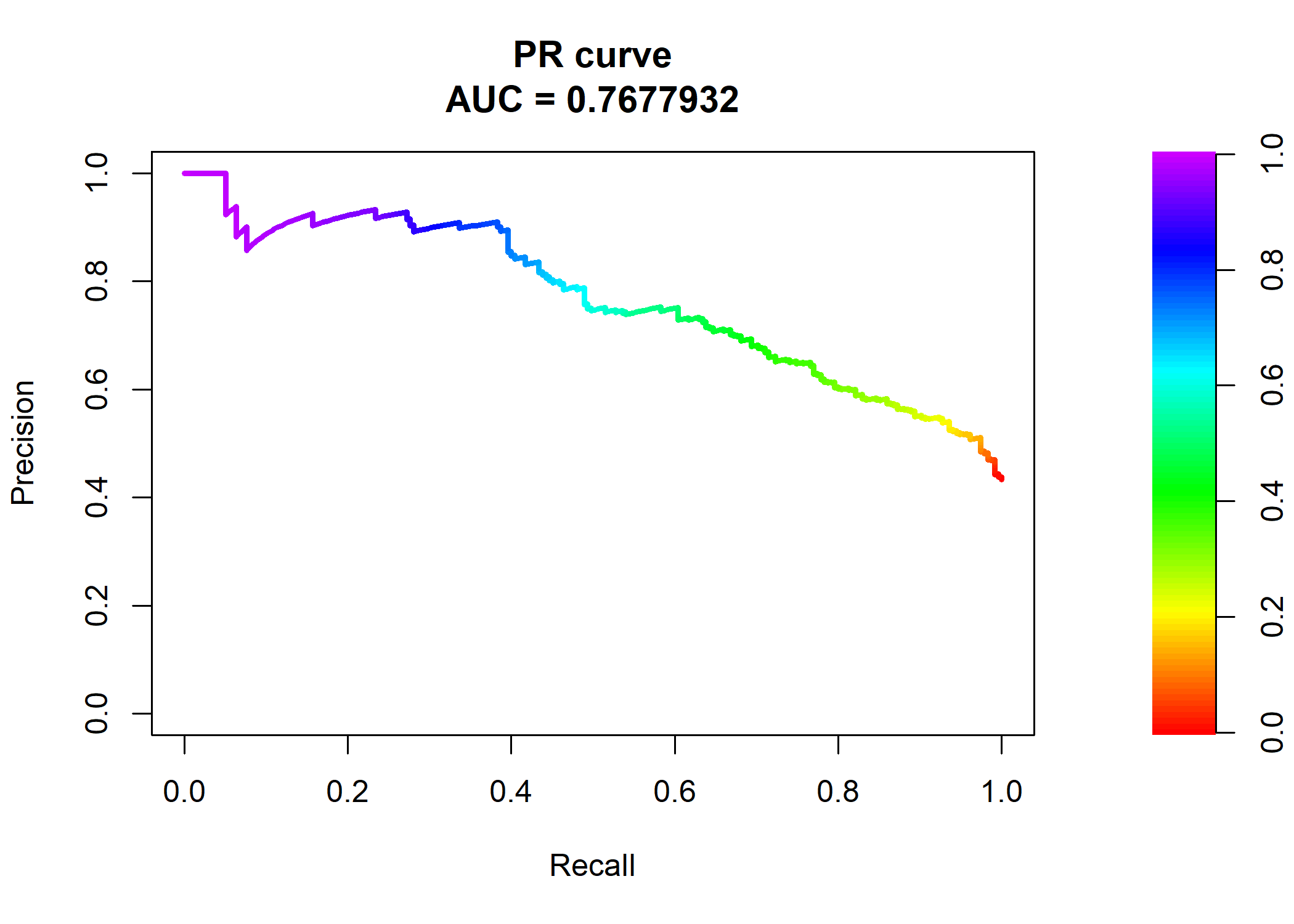

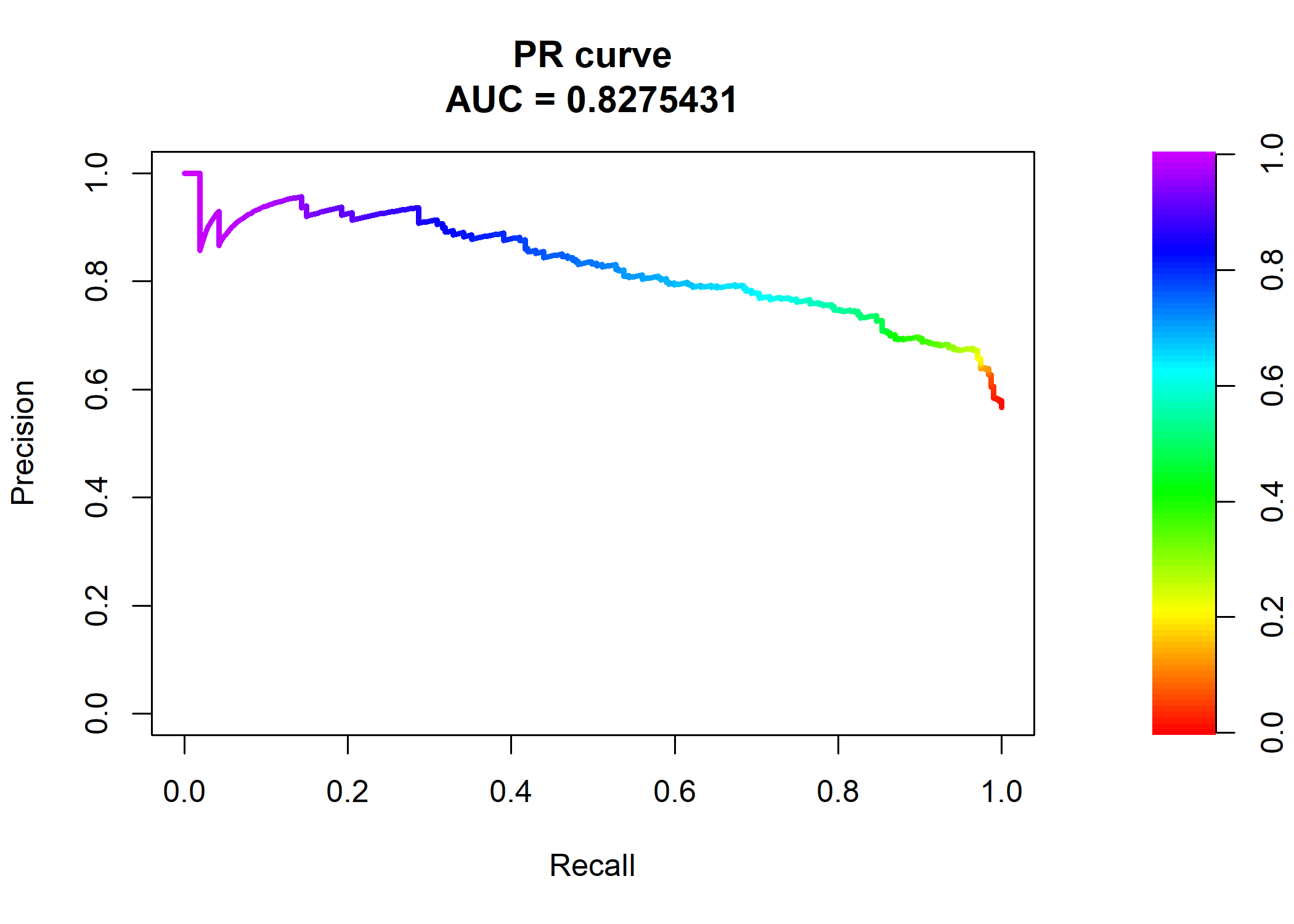


**C**

**D**

**Figure S3:** Precision response curves for Models 2-5. Colour scales represent the classification threshold for each point in the curve. A) Model 2 High-risk trajectory AUC = 0.718 B) Model 2 Low-risk trajectory AUC = 0.905 C) Model 3 High-risk trajectory AUC = 0.768 D) Model 3 Low-risk trajectory AUC = 0.828 E) Model 4 High-risk trajectory AUC = 0.781 F) Model 4 Low-risk trajectory AUC = 0.772 G) Model 5 High-risk trajectory AUC = 0.710 H) Model 5 Low-risk trajectory AUC = 0.905

**Secondary Outcome Definitions**

| **Outcome** | **Definition** |
| --- | --- |
| **Organ support** | Requirement for invasive mechanical ventilation, renal-replacement therapy or inotrope/vasopressor support |
| **Hospital length of stay** | Duration of whole hospital admission, measured in hours and reported as median (IQR 1-3) |
| **Hospital discharge disposition** | Discharge destination following hospital admission. They three destinations assessed were 1) home, 2) rehabilitation/other acute facility, 3) nursing home |
| **Annual trends in ICU LOS and bed occupancy** | For each study year, ICU median ICU was calculated.  ICU bed occupancy measured using two calculations:   1. Bed occupancy using median ICU LOS = Median ICU LOS x number of patients admitted for the specific year    1. Reported in days 2. ICU bed occupancy calculated by measuring cumulative ICU hours for the calendar year    1. Reported in days    2. Purpose of this measurement was to quantify the impact on bed occupancy by the outlier prolonged LOS cohort who are not captured by the median |

**References**

1. Gayat E, Cariou A, Deye N, Vieillard-Baron A, Jaber S, Damoisel C, et al. Determinants of long-term outcome in ICU survivors: results from the FROG-ICU study. Crit Care. 2018;22(1):8.

2. Braber A, van Zanten AR. Unravelling post-ICU mortality: predictors and causes of death. Eur J Anaesthesiol. 2010;27(5):486-90.

3. Fuchs L, Chronaki CE, Park S, Novack V, Baumfeld Y, Scott D, et al. ICU admission characteristics and mortality rates among elderly and very elderly patients. Intensive Care Med. 2012;38(10):1654-61.

4. Zimmermann T, Kaufmann P, Amacher SA, Sutter R, Loosen G, Merdji H, et al. Sex differences in the SOFA score of ICU patients with sepsis or septic shock: a nationwide analysis. Crit Care. 2024;28(1):209.

5. Modra LJ, Higgins AM, Pilcher DV, Bailey MJ, Bellomo R. Sex Differences in Mortality of ICU Patients According to Diagnosis-related Sex Balance. Am J Respir Crit Care Med. 2022;206(11):1353-60.

6. Muscedere J, Waters B, Varambally A, Bagshaw SM, Boyd JG, Maslove D, et al. The impact of frailty on intensive care unit outcomes: a systematic review and meta-analysis. Intensive Care Med. 2017;43(8):1105-22.

7. Sjögren L, Stenberg E, Thuccani M, Martikainen J, Rylander C, Wallenius V, et al. Impact of obesity on intensive care outcomes in patients with COVID-19 in Sweden-A cohort study. PLoS One. 2021;16(10):e0257891.

8. Sanaie S, Hosseini MS, Karrubi F, Iranpour A, Mahmoodpoor A. Impact of Body Mass Index on the Mortality of Critically Ill Patients Admitted to the Intensive Care Unit: An Observational Study. Anesth Pain Med. 2021;11(1):e108561.

9. Pando E, Alberti P, Mata R, Gomez MJ, Vidal L, Cirera A, et al. Early Changes in Blood Urea Nitrogen (BUN) Can Predict Mortality in Acute Pancreatitis: Comparative Study between BISAP Score, APACHE-II, and Other Laboratory Markers-A Prospective Observational Study. Can J Gastroenterol Hepatol. 2021;2021:6643595.

10. Liu Z, Meng Z, Li Y, Zhao J, Wu S, Gou S, et al. Prognostic accuracy of the serum lactate level, the SOFA score and the qSOFA score for mortality among adults with Sepsis. Scand J Trauma Resusc Emerg Med. 2019;27(1):51.

11. Oh TK, Song IA, Jeon YT. Peri-operative serum lactate level and postoperative 90-day mortality in a surgical ICU: A retrospective association study. Eur J Anaesthesiol. 2020;37(1):31-7.

12. Tan SC, Hayes L, Cross A, Tacey M, Jones D. Pre-medical emergency team activations - Patient characteristics, outcomes and predictors of deterioration. Aust Crit Care. 2023;36(6):1078-83.
